# Topological estimation of signal flow in complex signaling networks

**DOI:** 10.1038/s41598-018-23643-5

**Published:** 2018-03-27

**Authors:** Daewon Lee, Kwang-Hyun Cho

**Affiliations:** Department of Bio and Brain Engineering, Korea Advanced Institute of Science and Technology (KAIST), 291 Daehak-ro, Yuseong-gu, Daejeon, 34141 Republic of Korea

## Abstract

In a cell, any information about extra- or intra-cellular changes is transferred and processed through a signaling network and dysregulation of signal flow often leads to disease such as cancer. So, understanding of signal flow in the signaling network is critical to identify drug targets. Owing to the development of high-throughput measurement technologies, the structure of a signaling network is becoming more available, but detailed kinetic parameter information about molecular interactions is still very limited. A question then arises as to whether we can estimate the signal flow based only on the structure information of a signaling network. To answer this question, we develop a novel algorithm that can estimate the signal flow using only the topological information and apply it to predict the direction of activity change in various signaling networks. Interestingly, we find that the average accuracy of the estimation algorithm is about 60–80% even though we only use the topological information. We also find that this predictive power gets collapsed if we randomly alter the network topology, showing the importance of network topology. Our study provides a basis for utilizing the topological information of signaling networks in precision medicine or drug target discovery.

## Introduction

**Figure 1:**
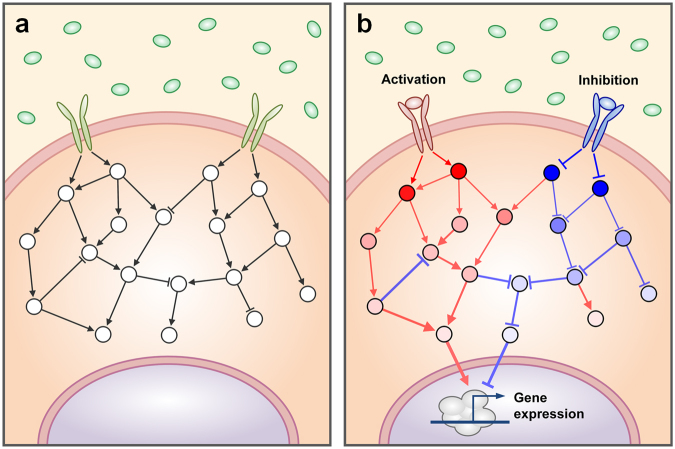
Illustration of a complex signaling network. (**a**) The topology of an exemplary signaling network. (**b**) The outcome of signaling. Red and blue links represent activating and inhibiting signals, respectively. Red and blue nodes indicate up-regulated and down-regulated nodes, respectively.

A cell processes any information about extra- or intra-cellular changes through a signaling network (Fig. 1a). In this process, critical information for cell fate determination such as survival, proliferation, differentiation, or death is transferred through a series of biochemical reactions, which can be defined as ‘signal flow’ in the signaling network (Fig. 1b)^1^. Dysregulation of the signaling network by a certain perturbation can lead to a fatal disease such as cancer since the altered signal flow might provide an incorrect information on the cell fate determination^2–4^. Hence, understanding of signal flow in complex signaling networks is critical to uncover the underlying mechanisms of the related disease and to identify promising drug targets.

Owing to the development of high-throughput measurement technologies, the topological information of signaling networks is becoming more available. For instance, Wang *et al*. reported a manually curated human signaling network composed of about 6,000 nodes and 63,000 links with the detailed regulatory information such as activation or inhibition^5,6^. In addition, KEGG (Kyoto Encyclopedia of Genes and Genomes) provides a knowledge base of almost 500 pathways containing important signaling pathways in cancer^7^.

However, detailed kinetic parameter information or logical relationships about molecular interactions for constructing mathematical models of complex signaling networks are still very limited. The construction of a rigorous mathematical model for a signaling network requires kinetic parameter values and the interaction logics as well as the information on network topology^8,9^. Even a very simplified network modeling such as Boolean network modeling requires a laborious process to collect the information on interaction logics or truth tables for the causal relationship between signaling molecules^10,11^. For differential equation modeling, a lot more experimental data and repetitive simulations are required to estimate the kinetic parameter values^12–16^.

A question then arises as to whether we can estimate the activity change of signaling molecules based only on the information of network topology. To address this question, we have developed a novel algorithm that can estimate the signal flow using only the topological information of signaling networks. The algorithm we developed aims to predict the direction of change in the activity of biomolecules (i.e., up or down) rather than predicting the accurate amount of the change.

We have applied our signal flow estimation algorithm to six signaling networks and found that it can properly estimate about 60–80% of signaling activity changes to all possible single or dual perturbations. We further found that the topological information of signaling networks is highly informative for predicting the activity changes by comparing the predictions to the cases of randomized network topologies. Our study is expected to provide a basis for utilizing the topological information of signaling networks in precision medicine or drug target discovery.

## Methods

### Signal propagation algorithm

**Figure 2:**
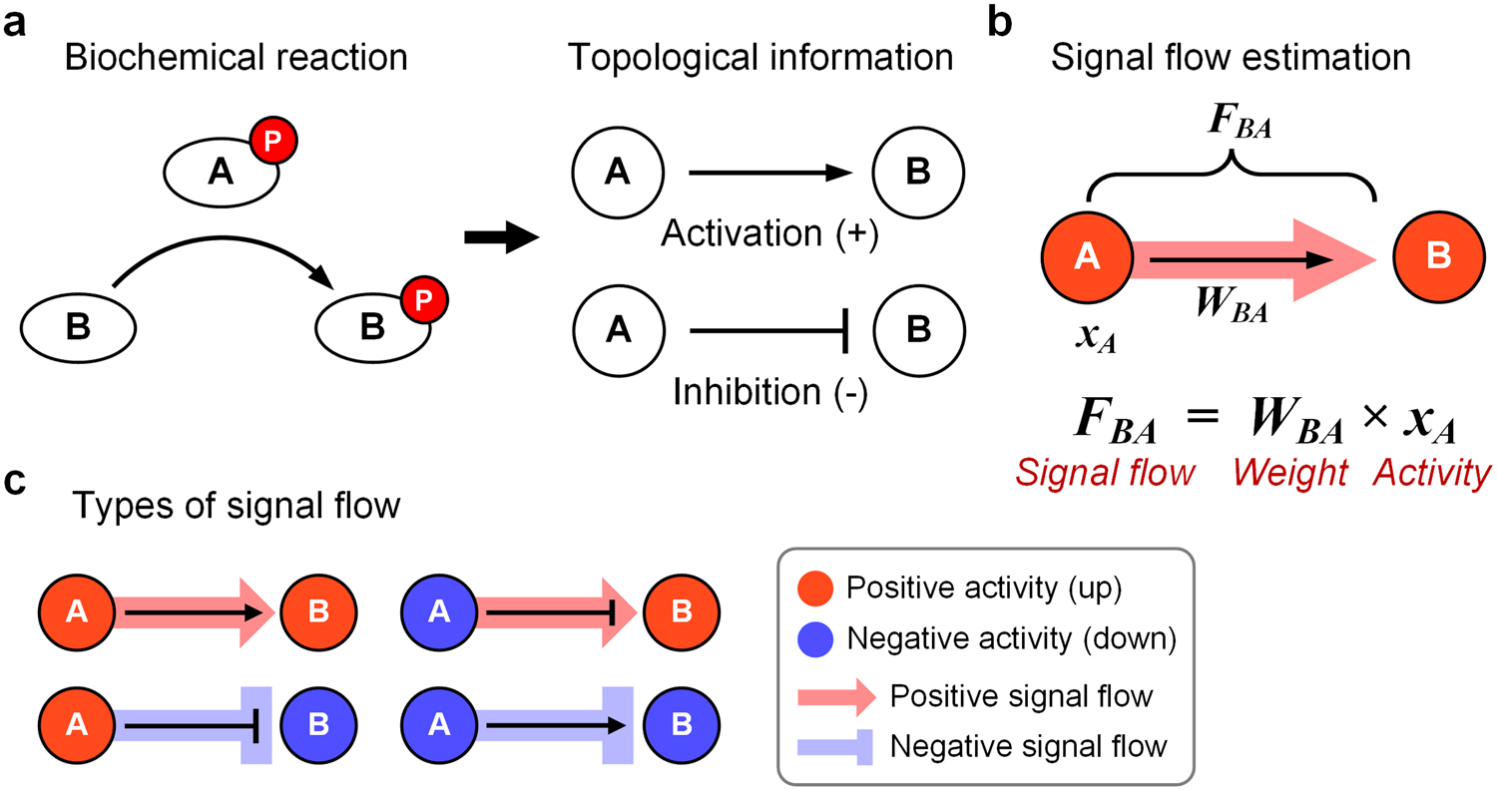
Topological information and signal flow. (**a**) A biochemical reaction such as phosphorylation of a protein in signaling networks can be represented by a directed link with a sign (i.e., a signed edge of a digraph). Activation and inhibition are denoted as plus (+) and minus (−) signs, respectively. **(b)** Signal flow is estimated by calculating the multiplication of the link weight and the activity of source node. **(c)** There are four types of signal flow. The sign of link and the sign of signal flow can be same or opposite depending on the source node activity and link weight where pointed arrow (→) indicates a positive weight and blunt arrow (⊣) denotes a negative weight.

The causal relationship between signaling molecules can be represented by a directed link with a sign in signaling networks, where the source (i.e., regulator) activates or inhibits the target through biochemical modification such as phosphorylation (Fig. 2a). The activity of a node can be mathematically defined as follows:

1 $${a}_{i}(t+1)={(\prod _{j}{a}_{j}{(t)}^{{W}_{ij}})}^{\alpha }{a}_{b}{(i)}^{1-\alpha },\alpha  \sim (0,1),$$

where *a*_*i*_(*t*) and *a*_*b*_(*i*) ∈ P are the activity at time *t* and the basal activity of node *i*, respectively. *W*_*ij*_ ∈ P is the weight of a link between node *j* and node *i*, which represents how much the node *j* affects the node *i* through the link. *α* ∈ P is a hyperparameter for the weighted multiplication, which has a range between 0 and 1. The equation (1) describes the activity of a node is determined by both the activities of its regulators and the basal activity of the node. We assumed that input stimulation does not change according to time for simplicity, and therefore the effect of input stimulation is reflected to the basal activity of input nodes. Taking the logarithm of equation (1), it becomes a linear difference equation as follows:

2 $$\begin{array}{rcl}{x}_{i}(t+1) & = & \alpha \sum _{j}{W}_{ij}{x}_{j}(t)+(1-\alpha ){b}_{i}\\ \to x(t+1) & = & \alpha Wx(t)+(1-\alpha )b\,(matrix\,notation)\end{array}$$

where *x* is *log*(*a*) ∈ P^*N*^, *b* is *log*(*a*_*b*_) ∈ P^*N*^, and *W* ∈ P^*N*×*N*^ is weight matrix. The equation (2) is the main formula of the signal flow estimation algorithm in this study, named ‘Signal Propagation (SP)’.

We can exactly solve the equation (2) at steady-state as follows:

$$\begin{array}{rcl}{x}_{s} & = & \alpha W{x}_{s}+(1-\alpha )b\\ \to {x}_{s} & = & (1-\alpha ){(I-\alpha W)}^{-1}b\end{array}$$

If the exact solution, *x*_*s*_, is not available, we can numerically solve it with an iterative method based on the equation (2). The iteration continues until a tolerance condition, ||*x*(*t* + 1) − *x*(*t*)|| < *tol* (e.g., *tol* = 10^−6^), is satisfied^17^. Note that the exact solution is not determined by the initial state of *x*, but by basal activity, *b*, that reflects the activity change caused by sustained stimulation or perturbation such as ligand binding, drug inhibition, or constitutively activating mutations.

### Topological estimation of signal flow

Signal flow is defined as how the activity of a signaling molecule influences the activity of another molecule through the link, and it is mathematically described as follows:

3 $${F}_{ij}(t)={W}_{ij}{x}_{j}(t),$$

where *F*_*ij*_ ∈ P is the signal flow, in which node *j* is the source and node *i* is the target (Fig. 2b**)**. The link weight, *W*_*ij*_, is determined by the topology of a given signaling network in the process of link weight normalization. Signal flows can be categorized into four types (Fig. 2c). The color of target node *B* denotes the effect of signal flow according to the definition of equation (3) (Fig. 2b). If source node *A* has a positive activity and link weight is positive, the signal flow is computed as positive according to the definition. Thus, node *B* receives a positive effect from this signal flow (Fig. 2c, top left). If source node *A* has a positive activity and link weight is negative, the signal flow is computed as negative according to the definition. Thus, node *B* receives a negative effect from this signal flow (Fig. 2c, bottom left). The signal flow from the down-regulated source node can be modelled by a positive signal flow that effectively up-regulates the target node^18,19^. In other words, a positive signal flow to the target is formed by the down-regulated activity of the source and its negative link to the target (Fig. 2c, top right). If source node *A* has a negative activity and link weight is positive, the signal flow is computed as negative according to the definition. Thus, node *B* is down-regulated by this signal flow (Fig. 2c, bottom right).

### Link weight normalization

**Figure 3:**
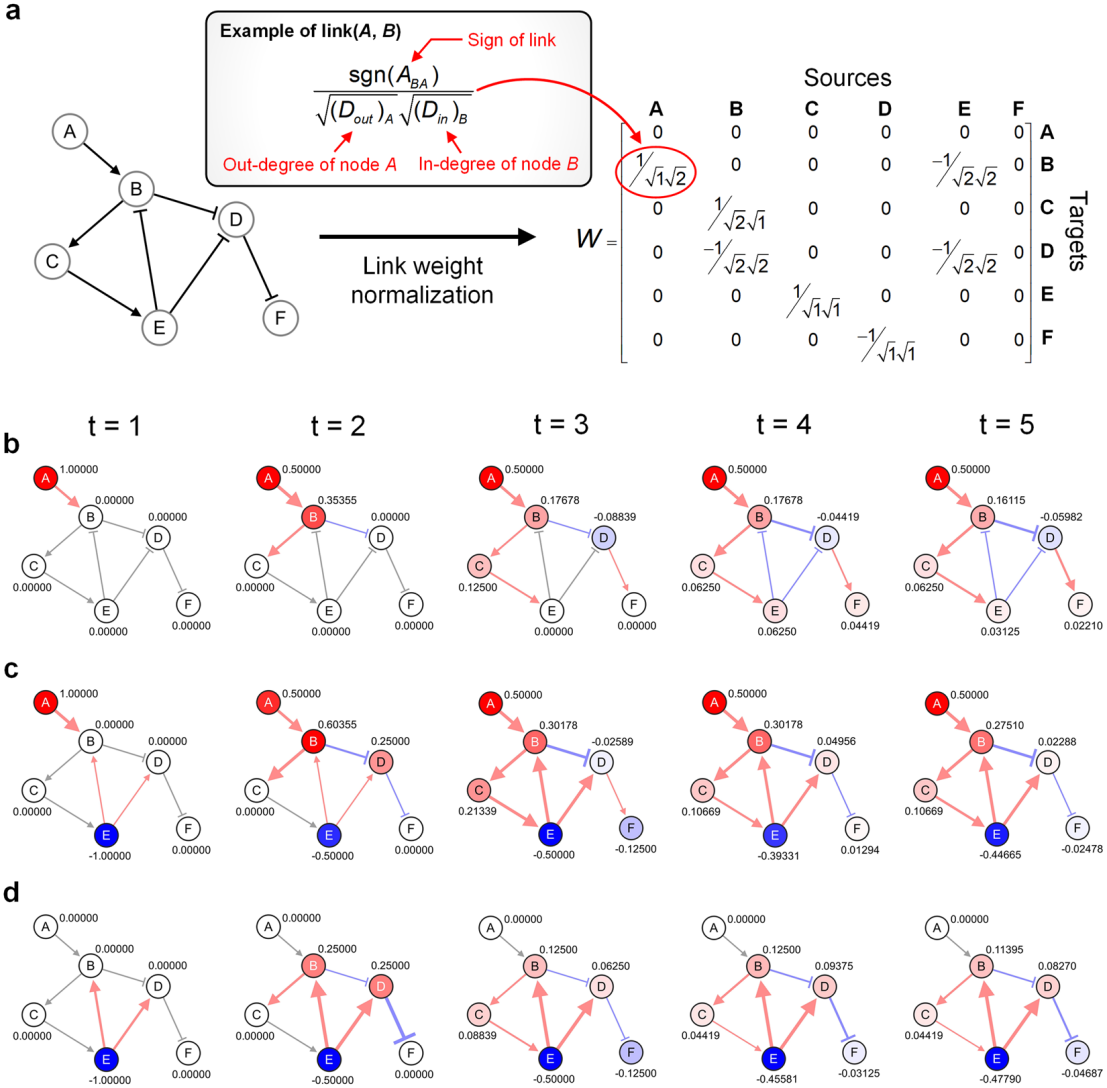
A toy example to explain how signal propagation algorithm works. (**a**) Toy example network and its link weight normalization. **(b–d)** The temporal evolution of activities and signal flows, **(b)** when node *A* is activated, or **(c)** when node *A* is activated and node *E* is inhibited. **(d)** The net effect of inhibiting node *E* calculated by comparing the results of (b) and (c). The real numbers in (b) and (c) denote the log-activity, *x*. The real numbers in (d) denote the difference between the log-activities of (b) and (c). The colors of nodes and links denote the relative quantity of the activities and signal flows. Red and blue colors of circles represent positive (up-regulated) and negative (down-regulated) activities, respectively. Red and blue colors of arrows denote positive (activating) and negative (inhibiting) signal flows, respectively. The values for basal activities of nodes *A* and *B* were assigned +1 and −1 (i.e., *b*_*A*_ =+1 and *b*_*B*_ = −1), respectively. The initial state, *x*(*t* = 1), was equal to the basal activity, *b* in each case, and the hyperparameter, *α*, is set to 0.5.

The link weight matrix, *W*, in equation (2) are normalized as follows:

4 $$W={D}_{in}^{-1/2}A{D}_{out}^{-1/2},{({D}_{in})}_{ii}=\sum _{j}|{A}_{ij}|,{({D}_{out})}_{jj}=\sum _{i}|{A}_{ij}|,$$

where *A* ∈ P^*N*×*N*^ is the adjacency matrix of a given network, and *D*_*in*_ and *D*_*out*_ are diagonal matrices for the in-degree and out-degree of the node, respectively (i.e., the summation of a column and the summation of a row, respectively). All weights consequently have decay types after the weight normalization. However, some link weights are not decay types if the source nodes have only one link at most. This weight normalization, together with hyperparameter *α*, not only prevents the divergence of the algorithm to obtain the solution of the equation (2)^20,21^, but also guarantees the predictive power of the algorithm to some extent. Figure 3a shows an example of the link weight normalization for a small toy network.

### Types of link weights

The link weights are divided into two types: (1) decay and (2) amplification. The absolute value of decaying weight is less than 1, which always makes the value of the signal flow, *F*_*ij*_, smaller than the activity of the source, *x*_*j*_, in equation (3). On the other hand, the absolute value of amplifying weight is greater than 1, which results in the amplified signal flow whose absolute value is greater than the activity of the source. However, if we introduce the link weight normalization, any type of link becomes effectively the decay type of link.

### A toy example of signal propagation algorithm

We present a toy example that demonstrates how SP algorithm works (Fig. 3b–d). Only node *A* has a change in its basal activity, while the other nodes have no change in their basal activities (Fig. 3b, *t* = 1). As the activity of node *A* propagates through its out-links, the activities of the other nodes are changed by the signal flows (Fig. 3b). In Fig. 3c, node *B* has a decrease in its basal activity (Fig. 3c, *t* = 1). Note that the signal flow can change its sign on the same link. For instance in Fig. 3b,c, the signal flows (i.e., *F*_*BE*_) at *t* = 4 have the opposite signs for the link between nodes *E* and *B*. This is because node *E* is up-regulated in Fig. 3b whereas it is down-regulated in Fig. 3c, and accordingly node *D* is oppositely regulated by node *E* in each case.

### Direction of activity change (DAC)

To analyze the net effect of input stimulations or perturbations, SP compares the results of different conditions by calculating the following difference.

5 $${x}^{fold}={x}^{{c}_{2}}-{x}^{{c}_{1}},$$

where *c*_*i*_ denotes each condition, not exponent. Equation (5) is basically a fold-change, since *x* is the logarithm of the activity. The sign of *x* reflects the direction of activity change (DAC), implying whether the signaling molecule is up-regulated or down-regulated. If the weight matrices of different conditions are not identical, signal flow should be estimated by considering the different weight matrices as follows:

6 $${{F}_{ij}}^{net}={W}_{ij}^{{c}_{2}}{x}_{j}^{{c}_{2}}-{W}_{ij}^{{c}_{1}}{x}_{j}^{{c}_{1}},$$

where the superscripts denote each condition, not exponent. In Fig. 3b,c, node *F* at *t* = 4 has positive activities in both conditions. However, node *F* is determined as down-regulated (Fig. 3d, the negative activity of node *F*) with respect to the perturbation of node *E*, as the signal flow from node *E* has a positive effect on node *D* in Fig. 3c. This emphasizes that the relative increase or decrease between the state variables of two conditions should be considered to interpret the DAC, rather than considering only the result of a single condition.

### Test datasets

We collected and curated 6 test datasets that are categorized into two types: ordinary differential equation model (**ODE**) and perturbation biology (**PB**) (Table 1). As there are few datasets in which both the activities of signaling molecules and network topology are available, we adopted the ODE models that are assumed to be reliable alternatives to reproduce the activities of signaling molecules. One of the advantages in utilizing a rigorously constructed ODE model is that we can perform simulations under a variety of conditions to avoid latent bias in the datasets. We generated 200, 80, and 48 sub-datasets (i.e., multiple data panels for the same simulation conditions) for B2009, S2011, and P2012, respectively, by varying simulation conditions such as input stimulation, the length of simulation time, and the type of activity measurement (i.e., the area under curve (AUC) or the concentration at the end of simulation as pseudo steady-state (SS)). Each data point of the generated datasets has the DAC information of the biomolecule. The directed networks of the ODE models were constructed by manually curating the biochemical reactions of the models. The active forms or functionally important forms of biomolecules were selected and merged to represent the nodes of directed networks. We also categorized the nodes into perturbation targets or readouts (i.e., targets to predict the DAC) according to their biological functions or topological aspects in the signal network. In the course of curating the network topologies, we did not optimize the topologies using the methods such as network inference algorithms. We allowed imperfect information from human mistakes in the curation to reflect real world situation.

**Table 1:** Data sets to test the algorithm.

Data	Size	N	L	I	P	R	C	Description
ODE	B2009^13^	22	47	2	13	13	66	ERK and AKT signaling pathways under EGF and insulin stimulations.
	S2011^45^	17	30	1	8	9	36	TNF related signaling pathways.
	P2012^46^	22	30	1	6	8	21	mTOR related signaling pathways.
PB	N2008^47^	15	24	1	6	9	21	ERK and AKT signaling pathways in MCF7 cells.
	M2013^19^	25	40	0	8	17	33	ERK and AKT signaling pathways in SKMEL-133 cells.
	K2015^18^	96	202	0	12	84	79	Large-scale signaling network in SKMel-133 cells.

**ODE:** ordinary differential equation model; **PB:** perturbation biology; **N**: number of nodes; **L**: number of links; **I**: number of inputs; **P**: number of perturbation targets; **R**: number of readouts; **C**: number of perturbation conditions.

Perturbation biology has been introduced by a series of studies from Sander and his colleagues, providing both protein activity data and the inferenced topological information of signaling networks (N2008, M2013, and K2015 in Table 1). Unlike the ODE datasets, PB datasets have only a single data panel that corresponds to a single sub-dataset of the ODE datasets. The data panel of a PB dataset consists of the log2-fold change of the activities of biomolecules, measured by western blot or reverse phase protein array (RPPA).

### The workflow for testing the algorithm

**Figure 4:**
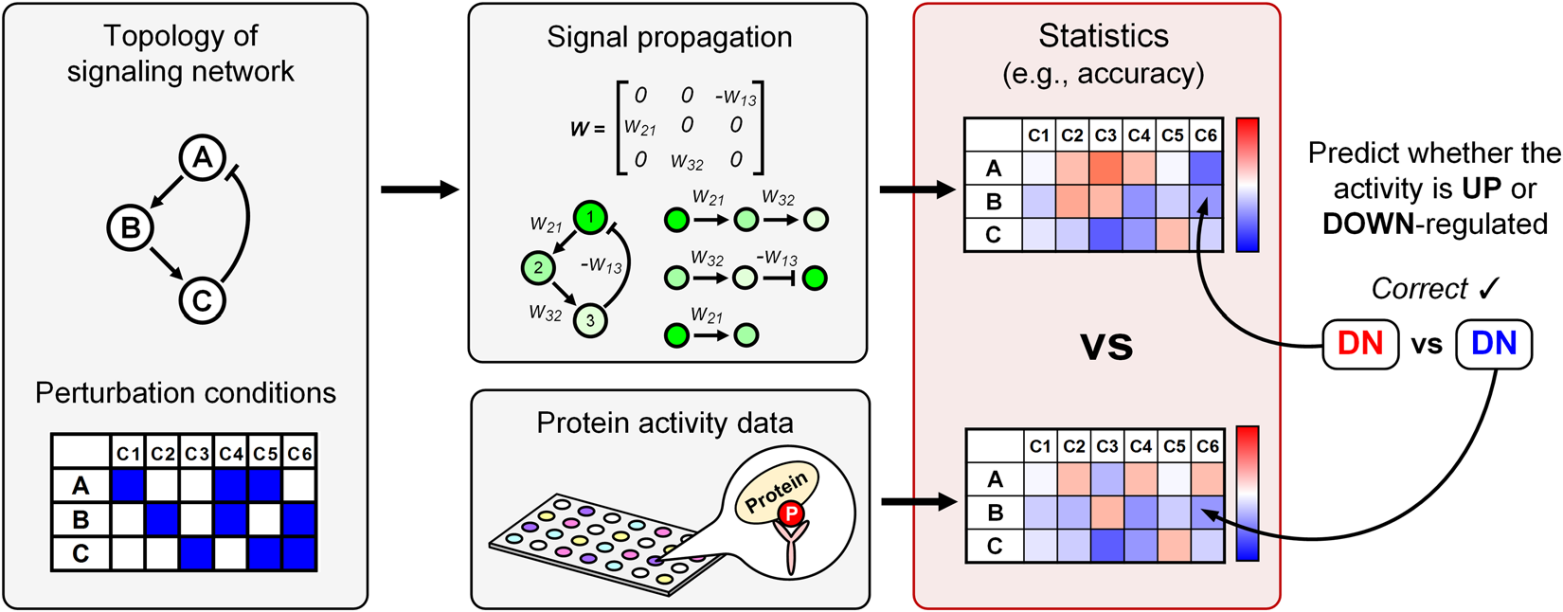
Workflow for testing the algorithm. A single dataset includes the topological information of a signaling network and perturbation conditions. Signal propagation algorithm estimates the signal flow using the network topology, and predicts the DAC. Accuracy is calculated as evaluation statistics by comparing the prediction results of the algorithm with the actual DAC of biomolecules. The hyperparameter, *α* = 0.5 was used and link weight normalization was applied, unless it is explicitly denoted.

We developed a workflow to test the predictive power of SP algorithm (Fig. 4). A single test dataset consists of (1) the topological information of a signaling network, (2) a panel of perturbation conditions, and (3) a panel of the actual DAC of biomolecules. SP algorithm is given both topological information and perturbation conditions, and it is tested for predicting the actual DAC of biomolecules.

The input stimulation or perturbation was reflected to the basal activity as explained in the toy example (Fig. 3). We assigned 1 and −1 as the values of basal activities for input stimulation and perturbation, respectively. Only negative values were used for perturbations, since all perturbations are inhibitory in all datasets.

The accuracy was calculated for each perturbation condition by comparing the DAC of SP algorithm and the actual DAC of test dataset. We calculated the prediction accuracy for each perturbation condition. For example, B2009 dataset consists of 200 sub-datasets, and a single sub-dataset includes 66 perturbation conditions for 13 readouts. So, in this case, we calculated the prediction accuracy for 200 × 66 = 13,200 times in total. However, since there is only one data panel in each of PB dataset, 33 accuracies, for example, can be calculated in M2013 dataset.

### Randomization of network topology

**Figure 5:**
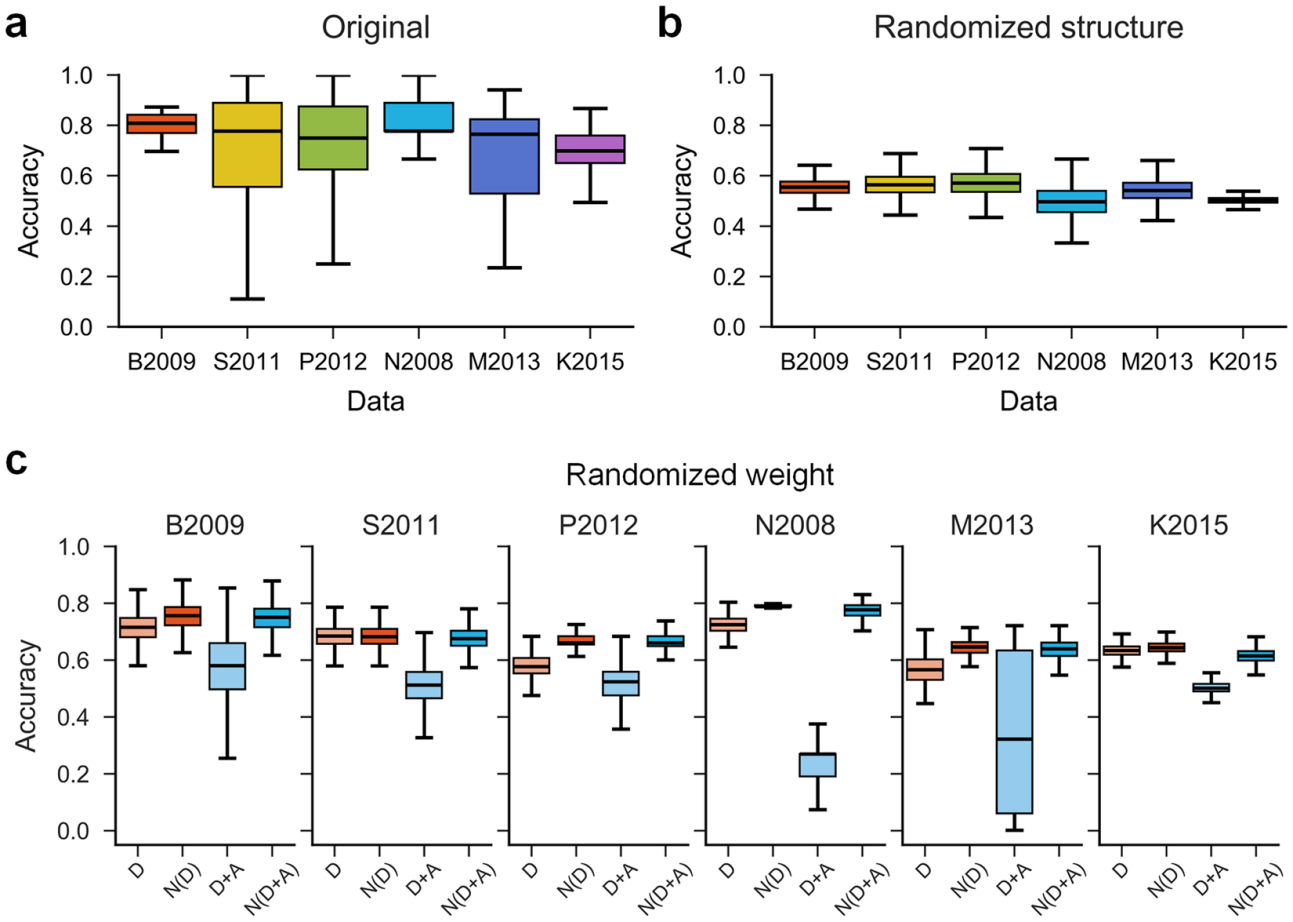
Overall accuracy of the algorithm. (**a**) The accuracies of predicting the DAC for the six datasets. **(b)** The accuracies of predicting the DAC under the degree-preserving randomization of network topology. In the randomization, the sign of a link is flipped or the targets of two links are swapped. **(c)** The accuracies of randomizing link weights based on the sampling policy. **D**: decay links only, weight ~ (0.001, 1); **D + A**: both decay and amplification links, weight ~ (0.001, 1000); **N**: link weight normalization is applied.

Randomizing the given network topology in Fig. 5 consists of link swapping and sign flipping. In link swapping, two links are randomly selected and the targets are exchanged between the two links. This way of link swapping ensures the preservation of degree distribution in the network. In sign flipping, a link is randomly selected and its sign is changed to the opposite one. For example, a positive link is changed to a negative link with the same source and target. We applied this randomization process repeatedly for a certain number of swappings and flippings. In this study, we set the number of swappings to the number of links and the number of flippings to the half of the number of links, respectively.

### Hierarchical clustering

**Figure 6:**
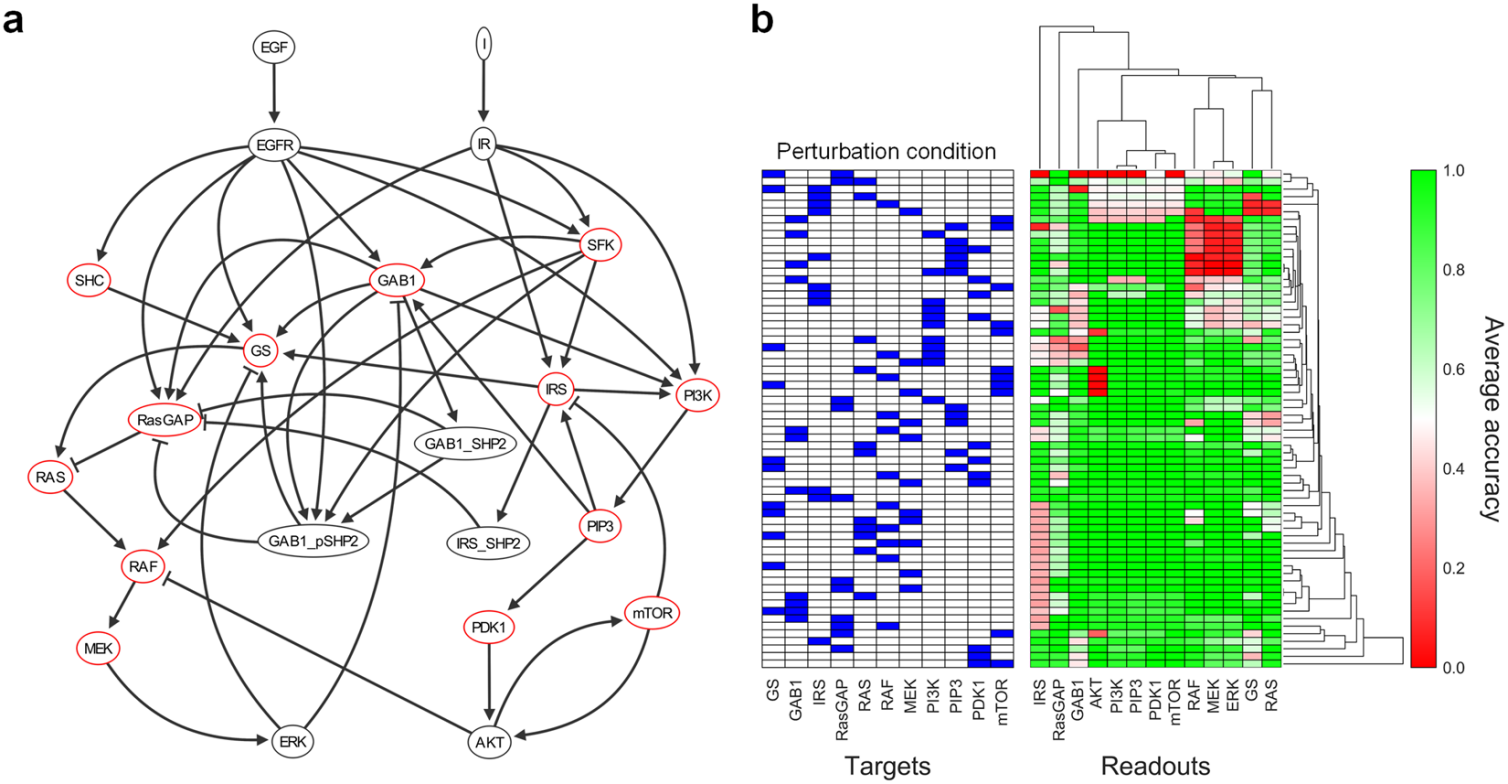
Network topology and hierarchical clustering result of B2009. (**a**) The network topology of B2009 where EGFR and IR signaling pathways are interconnected. Red nodes are the perturbation targets. **(b)** The hierarchical clustering of the average accuracies for the 200 sub-datasets of B2009. A single data element of the table represents the average accuracy for predicting the DAC of the readout across the 200 sub-datasets.

We performed hierarchical clustering for the results of the average accuracy (Fig. 6 and Supplementary Figs S3–S7) using the functionality of SciPy package (scipy.cluster.hierarchy)^22^. Manhattan distance was used in the pairwise distance calculation (scipy.spatial.distance.pdist) for creating the dendrograms of rows and columns in the hierarchical clustering.

### Data availability

We have created software libraries in Python programming language to efficiently analyze and visualize the estimated signal flows. The visualization software library fundamentally relies on PyQt (https://riverbankcomputing.com/software/pyqt). We provide a GitHub repository for the Python package to utilize the algorithm and datasets conveniently (https://github.com/dwgoon/sfa).

## Results

### The overall predictive power of SP algorithm

We applied SP algorithm to the six datasets (Table 1), and calculated the accuracy of predicting the DAC for each perturbation case. The accuracy is about 60–80% across the datasets (Fig. 5a). The minimum median accuracy is 0.678 of P2012, and the maximum median accuracy is 0.846 of B2009. The variation of accuracy is relatively larger for S2011, P2012, and M2013, whereas it is smaller for B2009, N2008, and K2015. Both the accuracy and the variation of each dataset show a moderate negative correlation with the number of nodes and the number of edges (Supplementary Fig. S1), implying that the predictive power of SP tends to decrease according to the size of signaling network.

To understand the role of the topological information in the prediction, we randomly generated an incorrect information of the topology, and calculated the accuracy of predicting the DAC. The accuracies of the six datasets are about 0.5–0.6 and the variations are small for the randomized topologies (Fig. 5b), implying that the incorrect information of topology makes the predictive power of SP algorithm almost close to random prediction.

We also randomized the link weights to explore the role of the weight in the algorithm. The policy of weight sampling was defined as four conditions: (1) decay (D; *w* ~ (0.001, 1)), (2) decay with weight normalization (N(A); *w* ~ (0.001, 1)), (3) decay + amplification (D + A; *w* ~ (0.001, 1000)), and (4) decay + amplification with weight normalization (N(D + A); *w* ~ (0.001, 1000)). The accuracies are about 60–80%, which are almost close to the original results, except the results of D + A (Fig. 5c). The cases of D + A for the six datasets have relatively lower accuracies and higher variations, suggesting the predictive power of SP becomes unreliable and unstable under the condition. On the other hand, the weight normalization for D + A condition recovered the predictive power of SP by forcing all the randomly sampled weights to have the decay type links. These results suggest that SP algorithm is basically more accurate and stable when it estimates signal flow based on the decay weight condition.

In addition, we analyzed the effect of hyperparameter, *α*. The condition that *α* is greater than 0.5 in equation (2) means the activity of a signaling molecule is more affected by signal flows than by basal activity. The hyperparameter, *α*, with a range of 0.1 to 0.9 has little effect on the overall predictive power of SP algorithm (Supplementary Fig. S2). Changing *α* does not substantially improve or weaken the predictive power of SP algorithm.

### Hierarchical clustering reflects the topological characteristics

The hierarchical clustering of the average accuracy shows concisely whether SP algorithm accurately predicts or not for each perturbation condition (Fig. 6 and Supplementary Figs S3–S7). Interestingly, the nodes that are adjacent in the topology of signaling network such as RAF-MEK-ERK cascade or PI3K-PIP3-PDK1 cascade in B2009 (Fig. 6a) are also closely located in the dendrogram of the hierarchical clustering (Fig. 6b). Pearson correlation between the distances of readout nodes in the dendrogram and the distances of nodes in the topology indicates moderate positive correlations for the six datasets (Supplementary Figs S8–S13). These results suggest the algorithm reflects the characteristics of topological information in the signal flow estimation. Especially, the prediction results of nodes within a simple linear path such as signaling cascade tend to be consistent with the result of the node at the highest position, suggesting that the algorithm reflects structural linearity and predicts the nodes as a whole in the linear structure.

### In-depth analysis of the hierarchical clustering result

**Figure 7:**
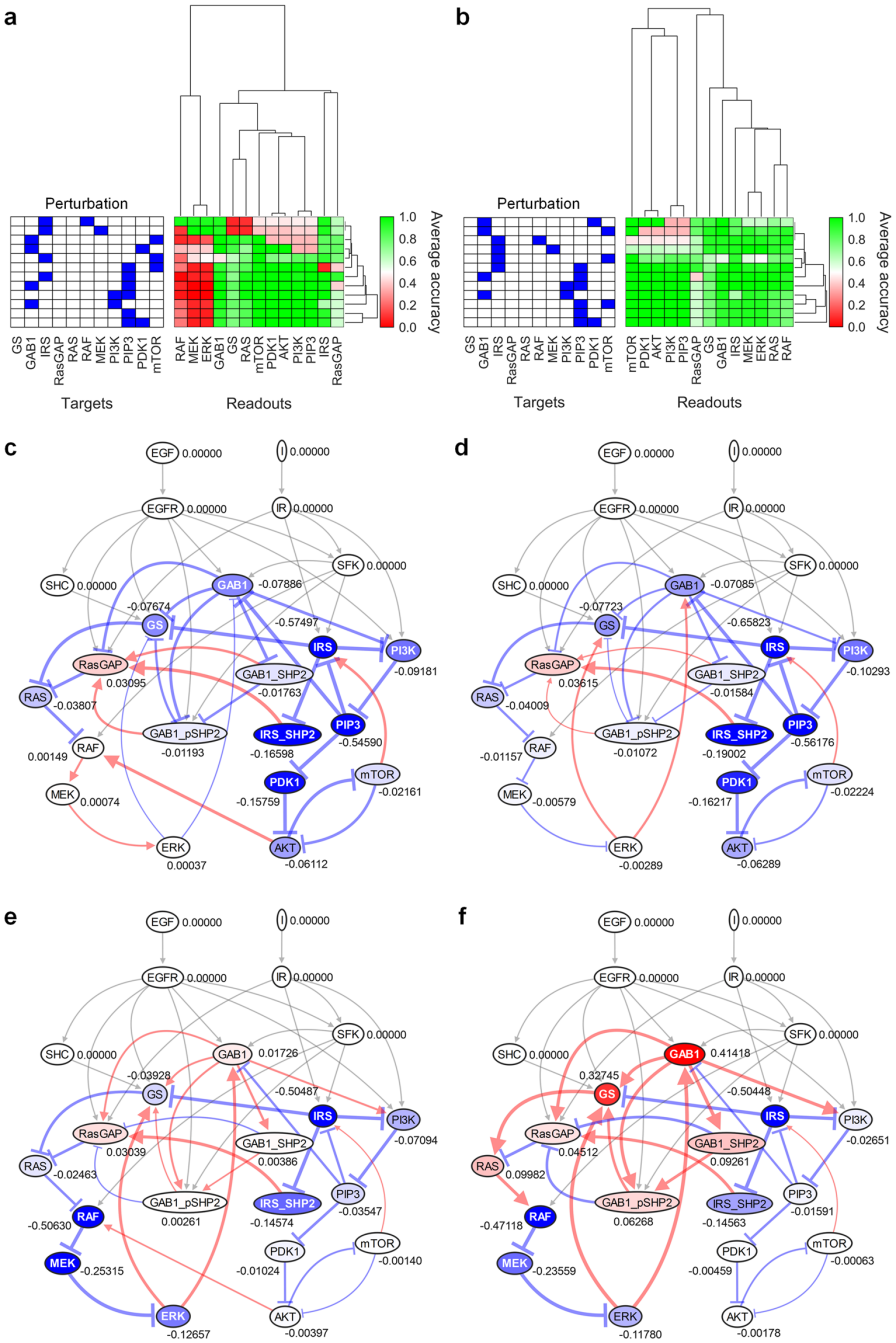
In-depth analysis of B2009. Perturbation conditions that SP algorithm failed to accurately predict GS-RAS-RAF-MEK-ERK cascade **(a**,**c**,**e)**. The modification of network topology (removal of the link between AKT and RAF) and the adjustment of the four link weights improved the results **(b**,**d**,**f)**. Signal flows and node activities are visualized for the perturbation of IRS and PIP3 (**c**,**d**) and the perturbation of RAF and IRS (**e**,**f**) before and after modifying the network topology and adjusting the link weights. In the adjustment of weights, the original weight values of four links (ERK to GS, ERK to GAB1, PI3K to PIP3, and PIP3 to IRS) were multiplied by 20, 20, 1.2, and 2, respectively after the weight normalization.

In order to gain a deeper understanding of SP algorithm, we analyzed the result of B2009 more specifically as a representative example (Fig. 7). The signaling network of B2009 consists of EGFR and IR pathways, in which RAS-RAF-MEK-ERK signaling cascade and PI3K-PIP3-PDK1-AKT signaling cascade are interconnected by various crosstalks, forming a complex signaling network (Fig. 6a).

First, we analyzed the cases where SP algorithm failed to predict correctly. SP algorithm was incorrect for RAF, MEK, and ERK to the perturbations including PI3K, PIP3, or PDK1 (Fig. 6b, red area for RAF, MEK, and ERK). SP algorithm also did not succeed in predicting the DAC of GS and RAS for the perturbation of IRS and RAF, and the perturbation of IRS and MEK (Fig. 6b, red area for GS and RAS). We conjectured the failure of the algorithm for these cases would be due to incorrect information on the network topology or the decaying aspect of link weights. Since the inhibition of RAF by AKT was very weak in HEK293 cells, the inhibitory reaction was negligible in the original ODE model of B2009^13^. In addition, the attenuation of signal flow under the decay weight condition was considered as a major cause of the failure in predicting GS and RAS. Therefore, we removed the link between AKT and RAF, and adjusted some weights to amplify signal flows in the signaling network. These modifications on the topology and link weights improved the accuracies of GS, RAS, RAF, MEK, and ERK (Fig. 7a,b). Under the perturbation of IRS and PIP3, the positive signal flow through the link between AKT and RAF up-regulated RAF-MEK-ERK cascade (Fig. 7c), whereas RAF-MEK-ERK cascade decreased without the link (Fig. 7d). In the case of the perturbation of IRS and RAF, GS and RAS were down-regulated due to the decay weights (Fig. 7e), whereas GS and RAS were up-regulated when we amplified signal flows by adjusting four weights: the negative feedbacks of ERK (ERK to GS and ERK to GAB1), and AKT cascade (PI3K to PIP3 and PIP3 to IRS) (Fig. 7f). Adjusting more weights achieved complete agreement between the results of SP and the original ODE model in predicting the DAC (Supplementary Figs S14, S15). Another example of B2009 also shows adjusting weights improved the accuracy of predicting the DAC of AKT under the perturbation of mTOR, and achieved the complete agreement (Supplementary Fig. S16).

## Discussion

The topological properties of complex networks were explored under various contexts including scale-freeness, controllability, observability, *etc*.^23–27^. Considering that the topology of complex networks conveys significant information, we attempted to estimate the signal flow in a cell signaling network based only on the topological information even though the signal flow is actually determined by network dynamics that depends on both topology and kinetic parameter values. Intriguingly, our signal flow estimation algorithm could predict the signal flow change of various signaling networks with 60–80% of accuracy. Such prediction was, however, easily disrupted if we randomize any part of the network topology, which indicates that the topology of a signaling network has been refined over and over during evolution and conveys critical information of signal flow.

There were some related studies with different purposes and scopes (see Supplementary Methods for details). Feiglin *et al*. proposed a possibility to predict the phenotypic effects of perturbations based on the static network structure of regulatory networks^28^. Arakelyan and his colleagues proposed an algorithm to estimate signal flow in pathways^29,30^, and elucidated molecular mechanisms underlying malignant and chronic lung diseases by integrating the algorithm with gene expression data^31^. A set of algorithms based on Gaussian smoothing^32^ was proposed for unsigned^33,34^ or undirected^21^ networks to predict gene functions^33^ or mutational effects^35,36^.

The proposed approach has still many limitations to be overcome. We found that sometimes an incorrect information on a single link can result in a significant failure in predicting the signal flow changes in multiple adjacent nodes in the network as can be seen in the case of a link between AKT and RAF in B2009. So, we need to make sure that the topology of a given signaling network is accurate enough to avoid such critical failure before we apply the proposed approach to estimate signal flow change in the network. To improve or supplement the accuracy of network topology, we can employ data-driven network inference/curation algorithms^37–39^. On the other way around, we can use the proposed algorithm to find out the critical parts of a signaling network and use this information to control the overall signal flow in the network. Another limitation of the proposed algorithm is finding out optimal link weights for implementation of the algorithm. Although there were some studies investigating the geometric nature of weights in complex networks^40^, it is still hard to find out optimal weights that are most pertinent to our algorithm. A similar issue was raised that information on system dynamics as well as network topology is required to identify an optimal set of nodes to be controlled to regulate the dynamics of a biological network in a desired direction^41^. One possible way of dealing with this problem is integrating both topological information and high-throughput measurement data for gene expression or protein activity^31,42,43^.

The topological information of biological networks is rapidly growing. So, the proposed signal flow estimation approach can be widely used along with the increase of such information. In particular, the proposed algorithm can be applied to finding out control target nodes/links for drug target discovery, drug repositioning, and precision medicine. We expect our approach will affect a wide range of applications that utilize the abundant topological information of biological networks. A signal flow estimation algorithm improved to obtain higher predictive power can be exploited to discover the control targets in signaling networks, which are the candidates of drug target for cancer treatment^44^. Ultimately, it is possible to establish one of the important foundation works for implementing precision medicine by deciphering the topological information of biological networks from patients.

## Electronic supplementary material

Supplementary Information
