# Supplementary material for: Topological estimation of signal flow in complex signaling networks
Source: Sci Rep. 2018 Mar 27;8:5262. doi: 10.1038/s41598-018-23643-5 (PMC5869720; doi:10.1038/s41598-018-23643-5)
Supplement: Supplementary file 1 — Supplementary Information [file 41598_2018_23643_MOESM1_ESM.pdf]

Supplementary Information

Topological estimation of signal flow in complex signaling networks

Daewon Lee and Kwang-Hyun Cho*

Department of Bio and Brain Engineering,

Korea Advanced Institute of Science and Technology (KAIST),

291 Daehak-ro, Yuseong-gu, Daejeon, 34141, Republic of Korea

*Corresponding author. E-mail: ckh@kaist.ac.kr, Phone: +82-42-350-4325, Fax: +82-42-350-4310, Web: http://sbie.kaist.ac.kr/

Contents

I. Suppplementary Methods

S1. Relationships with other algorithms

S1.1. Acyclic Path Summation (APS)

S1.2. Cyclic Path Summation (CPS)

S1.3. Pathway Signal Flow (PSF)

S1.4. Gaussian Smoothing (GS)

II. Supplementary Figures

Supplementary Figure S1. Correlation between accuracy and network size.

Supplementary Figure S2. The effect of hyperparameter, α.

Supplementary Figure S3. Network structure and hierarchical clustering result of S2011.

Supplementary Figure S4. Network structure and hierarchical clustering result of P2012.

Supplementary Figure S5. Network structure and hierarchical clustering result of N2008.

Supplementary Figure S6. Network structure and hierarchical clustering result of M2013.

Supplementary Figure S7. Network structure and hierarchical clustering result of K2015.

Supplementary Figure S8. Correlation between the distances in network structure and the distances in dendrogram of hierarchical clustering for B2009.

Supplementary Figure S9. Correlation between the distances in network structure and the distances in dendrogram of hierarchical clustering for S2011.

Supplementary Figure S10. Correlation between the distances in network structure and the distances in dendrogram of hierarchical clustering for P2012.

Supplementary Figure S11. Correlation between the distances in network structure and the distances in dendrogram of hierarchical clustering for N2008.

Supplementary Figure S12. Correlation between the distances in network structure and the distances in dendrogram of hierarchical clustering for M2013.

Supplementary Figure S13. Correlation between the distances in network structure and the distances in dendrogram of hierarchical clustering for K2015.

Supplementary Figure S14. Adjustment of link weights and the result of signal flow estimation.

Supplementary Figure S15. The time profiles of the original ODE model under the perturbation of IRS and RAF.

Supplementary Figure S16. An example of prediction result improved by adjusting link weights.

III. Supplementary References

I. Supplementary Methods

S1. Relationships with other algorithms

We describe the relationships of signal propagation (SP) with other algorithms such as pathway signal flow (PSF), acyclic and cyclic path summation (APS and CPS), and Gaussian smoothing (GS). We explain similar or different aspects of the algorithms under particular conditions. The relationships are summarized as follows.

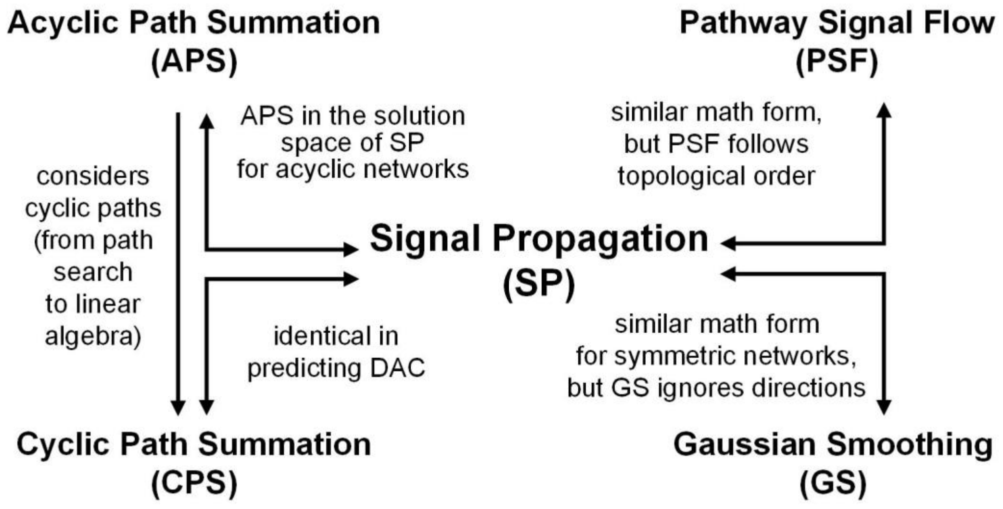

S1.1. Acyclic Path Summation (APS)

Feiglin *et al.* demonstrated a possibility to predict the phenotypic effects of perturbations using only the static structure of regulatory networks [1]. We here show the solution of Feiglin *et al.* is in the solution space of SP algorithm. We use a different notation rather than the original notation of Feiglin *et al.* for the purpose of clarifying our logic.

Definition 1. $F^L \in \mathbb{R}^{N\times N}$, the influence matrix that describes the influence of a source node *j* on a destination node *i* through all simple paths with length *L*, is defined as

$$F_{ij}^{L} = \sum W_{k_1 j} W_{k_2 k_1} \cdots W_{k_{L-1} k_{L-2}} W_{i k_{L-1}}\ ,\ \ F^0 = I\ ,\ \ F^1 = W\ , \qquad \textbf{(S1)}$$

where $W \in \mathbb{R}^{N\times N}$ is the matrix of link weights, and the sequence, $(j, k_1, k_2, \ldots, k_{L-1}, i)$, is a simple path from node *j* to node *i*. The above *F* is not the matrix of signal flow in the main manuscript. The superscript of *F* in equation (**S1**) is the path length, *L*, not the exponential notation of matrix.

Definition 2. $E^L \in \mathbb{R}^{N\times N}$, the total effect of signaling sources on node *i* through all paths with length *L*, is defined as

$$E_i^L = \sum_{j=1}^{N} F_{ij}^L \cdot b_j \rightarrow E^L = F^L \cdot b, \qquad \textbf{(S2)}$$

and the combined effect (C) of all paths with any length is defined as

$$C = \sum_{L=1}^{L_M} E^L, \qquad \textbf{(S3)}$$

where L_M is the longest path length, and $b \in \mathbb{R}^N$ is the basal activity or signaling source.

Lemma 1. *For any path length* $L \in \mathbb{N}$ *in a directed acyclic graph (DAG),*

$$F^L = W^L.$$

Proof. We will prove by induction that, for all $n \in \mathbb{N}$,

$$F^n = W^n \qquad \textbf{(S4)}$$

Note that n of F in equation (**S4**) is not exponent as in the equation (**S1**) of **Definition 1**.

Base case:

If $n = 1$,

$$F^1 = W$$

Thus, equation (**S4**) is true for $n = 1$.

If $n = 2$,

$$F_{ij}^2 = \sum_{k=1}^{N} W_{ik} F_{kj}^1 \quad \rightarrow \quad F^2 = WF^1 = W^2 \qquad \textbf{(S5)}$$

which demonstrates equation (**S4**) is true for $n = 2$.

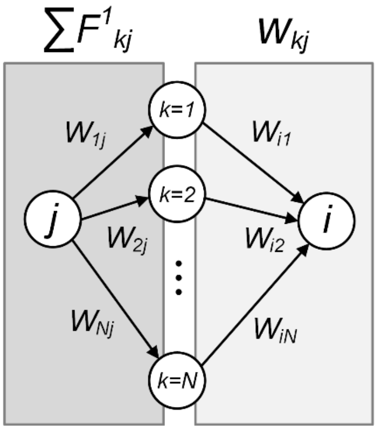

The visualization of equation (**S5**). To expand the simple paths from node j by one length, all links that connect source node j in F^1 to the destination node i are included.

Induction step:

Let $l \in \mathbb{N}$ be given and suppose equation (**S4**) is true for $n = l$. Then,

$$\begin{aligned} F^{l+1} &= \sum_{k=1}^{N} W_{ik} F^{l}_{kj} \qquad (\textit{entry notation}) \\ &= WF^{l} \\ &= WW^{l} \qquad (\textit{by induction hypothesis}) \\ &= W^{l+1} \quad . \end{aligned}$$

Thus, equation (**S4**) holds for $n=l+1$, and the proof of the induction step is complete.

By the principle of induction, equation (**S4**) is true for all $n \in \mathbb{N}$. □

Corollary 1. *Lemma 1 is basically the same as the proof of the theorem that the* (i, j)*-entry of the matrix* A^L *is equal to the number of walks from vertex i to vertex j in the graph of length* L^2.

Remark 1. The proof of lemma 1 shows F^L can be equal to W^L, if the network is a **DAG**. For cyclic networks, W^L includes all **walks**, thus, $F^L \neq W^L$.

We consider the relationship between APS and SP algorithm for acyclic networks, where any cycle does not exist. At first, we will show the combined effect, C, of APS can be represented by a linear combination of vectors, if a network has no cycle. Then, we will demonstrate the solution of APS is in the solution space of SP. The combined effect with the longest path length, L, in APS can be described by

$$
\begin{aligned}
C_L &= E^0 + E^1 + E^2 + \cdots + E^L \\
&= (F^0 + F^1 + F^2 \cdots + F^L)b \\
&= (I + W^1 + W^2 \cdots + W^L)b \\
&= (I + wA^1 + w^2A^2 + \cdots + w^L A^L)b,
\end{aligned} \quad \textbf{(S6)}
$$

where A is the adjacency matrix with signs, w is the global link weight, and b is the signaling source. The global link weight is the weight value that Feiglin *et al.* used to assign all links the same value (e.g., 0.5).

SP algorithm can be described with the log-activity, x, at time t as follows.

$$x(t) = \alpha^t W^t b + (1-\alpha)(I + \alpha W + \alpha^2 W^2 + \cdots + \alpha^{t-1} W^{t-1})b, \quad \textbf{(S7)}$$

where W and α is the same as those in the main manuscript. This is the explicit form, while the equation (2) in the main manuscript is a difference equation form.

Theorem 1. *The solution of C_L is in the solution space of $x(t)$, if a given network has no cycle, and signal is assumed to attenuate during propagation.*

Proof. If we replace W, α, and t with A, w, and L, respectively, equation (**S7**) becomes as follows.

$$x(L) = w^L A^L b + (1-w)(I + wA + w^2A^2 + \cdots + w^{L-1}A^{L-1})b, \quad \textbf{(S8)}$$

where $|w| < 1$. Subtracting equation (**S8**) from equation (**S6**) results in the relationship between C_L and $x(L)$ as follows.

$$
\begin{aligned}
C_L - x(L) &= w(I + wA + w^2A^2 + \cdots + w^{L-1}A^{L-1})b \\
C_L - x(L) &= wC_{L-1} \\
C_L &= wC_{L-1} + x(L).
\end{aligned} \quad \textbf{(S9)}
$$

Equation (**S9**) is a recurrence relation problem, and the solution is

$$
\begin{aligned}
C_L &= wC_{L-1} + x(L) \\
\frac{C_L}{w^L} &= \frac{C_{L-1}}{w^{L-1}} + \frac{x(L)}{w^L} \\
\frac{C_L}{w^L} &= \frac{C_0}{w^0} + \sum_{k=1}^{L} \frac{x(k)}{w^k} \\
C_L &= x(0) + w^{L-1}x(1) + w^{L-2}x(2) + \cdots + x(L),
\end{aligned} \tag{S10}
$$

where C_0 is $b{=}x(0)$. Equation (**S10**) shows C_L can be represented by a linear combination of the solutions of $x(t)$, meaning C_L is in the solution space of $x(t)$. □

Remark 2. The equation (**S9**) can also be described by a matrix-vector multiplication, in which $x(L)$ is assumed not to change with respect to L for convenience.

$$
\begin{bmatrix} 1 & -1 \\ 0 & 1 \end{bmatrix}\begin{bmatrix} (C_L)_i \\ (x(L))_i \end{bmatrix} = \begin{bmatrix} w & 0 \\ 0 & 1 \end{bmatrix}\begin{bmatrix} (C_{L-1})_i \\ (x(L-1))_i \end{bmatrix}
$$

$$
\to \begin{bmatrix} (C_L)_i \\ (x(L))_i \end{bmatrix} = \begin{bmatrix} w & 1 \\ 0 & 1 \end{bmatrix}\begin{bmatrix} (C_{L-1})_i \\ (x(L-1))_i \end{bmatrix}
$$

$$
\to u_L = Ru_{L-1},
$$

where $(C_L)_i$ and $(x(L))_i$ are the i-th elements of C_L and $x(L)$, respectively. R, the relationship matrix, is diagonalizable, if one of the eigenvalues, w, is not 1.

$$
\begin{aligned}
R &= S\Lambda S^{-1} \\
&= \begin{bmatrix} 1 & 1 \\ 0 & 1-w \end{bmatrix}\begin{bmatrix} w & 0 \\ 0 & 1 \end{bmatrix}\begin{bmatrix} 1 & \dfrac{-1}{1-w} \\ 0 & \dfrac{1}{1-w} \end{bmatrix}.
\end{aligned}
$$

Thus, the analytic solution of u_L is

$$
\begin{aligned}
u_L &= R^L u_0 \\
&= S\Lambda^L S^{-1} u_0
\end{aligned}
$$

$$= S\begin{bmatrix} w^L & 0 \\ 0 & 1 \end{bmatrix} S^{-1}u_0 \qquad \textbf{(S11)}$$

$$= \begin{bmatrix} w^L C_0 + \dfrac{1-w^L}{1-w} x_0 \\ x_0 \end{bmatrix}. \qquad \textbf{(S12)}$$

The first element of equation (**S12**) is the same as that of equation (**S10**). With the diagonalized R^n, equation (**S11**) shows us the stability of C_L depends on w, which is usually set to be less than 1.

Example 1. Consider a two-node negative feedback loop. The combined effect, C_L, under the perturbation on node (*1*) can be calculated as follows.

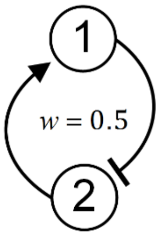

$$A = \begin{bmatrix} 0 & -1 \\ 1 & 0 \end{bmatrix}$$

$$b = \begin{bmatrix} -1 \\ 0 \end{bmatrix}$$

$$C_1 = (I + wA)b = \begin{bmatrix} -1 \\ -0.5 \end{bmatrix},$$

where L is 1, because the longest acyclic path length is 1. C_L also can be obtained from the solution space of x.

$$\begin{aligned} C_1 &= wC_0 + x_1 = wb + x_1 \\ &= wb + wAx(0) + (1-w)b = wAb + b = (wA + I)b \\ &= \begin{bmatrix} 0.5 & -1 \\ 1 & 0.5 \end{bmatrix}\begin{bmatrix} -1 \\ 0 \end{bmatrix} = \begin{bmatrix} -1 \\ -0.5 \end{bmatrix}. \end{aligned}$$

On the other hand, the steady-state solution of x is

$$x(\infty) = (1-\alpha)(I-\alpha W)^{-1}b$$
$$= \frac{1}{3}\begin{bmatrix} -1 \\ -0.5 \end{bmatrix}.$$

Note that the signs of C_L and x (i.e., DACs of C_L and x) coincide.

S1.2. Cyclic Path Summation (CPS)

Cyclic path summation (CPS) is a derivative of APS algorithm, which includes cyclic paths (cycles). The length of a cyclic path can be considered as infinite, if the cyclic paths are counted repeatedly. Thus, the combined effect is defined as

$$C = \sum_{L=0}^{\infty} E^L = \sum_{L=0}^{\infty} F^L \cdot b$$
$$= \sum_{L=0}^{\infty} W^L \cdot b\,, \qquad \textbf{(S13)}$$

where b, F, E, and W are the same as those of APS in **S1.1. Acyclic Path Summation (APS)** .

The combined effect of CPS is the same as equation (**S6**).

$$C_\infty = (I + W + W^2 + \cdots + W^\infty)b\,. \qquad \textbf{(S14)}$$

The steady-state solution of equation (**S7**) is as follows.

$$x(\infty) = (1-\alpha)(I + W + W^2 + \cdots + W^\infty)b\,. \qquad \textbf{(S15)}$$

Equation (**S15**) is equal to $(1-\alpha)$ times equation (**S14**), which means CPS and SP have the same results for predicting the DAC of steady-state solution, because $(1-\alpha) \geq 0$ does not affect the sign of both C_∞ and $x(\infty)$. Feiglin *et al.* implemented APS based on path searching, excluding cycles [1], but it is possible to implement CPS more efficiently based on linear algebra like the implementation of SP (refer to the **Methods** section in the main manuscript).

S1.3. Pathway Signal Flow (PSF)

Arakelyan and his colleagues have developed Pathway Signal Flow (PSF) algorithm to estimate signal flow in pathways [3,4], and applied PSF algorithm to elucidate molecular mechanisms underlying malignant and chronic lung diseases [5]. PSF share similar concepts for signal flow with SP, but the application of PSF is integrated with high-throughput data such as gene expression or protein activity [5].

Nersisyan *et al.* implemented a Cytoscape plugin, named PSF Calculator (PSFC), to facilitate more generalized usage of the algorithm (http://apps.cytoscape.org/apps/psfc) [4]. In PSFC, the definitions of mathematical functions are required to describe how signal flow is calculated according to the interaction type [4]. The following is the description of SP algorithm in the style of PSFC rules.

Activation (*)	source * target
Inhibition (/)	1/(source * target)
Multiple input signals	Multiplication
Splitting	Normalization by in- and out-degrees

$$a_i = \left(a_i \prod_{j \in activators} a_j^{|w_{ij}|} \right) \left(\frac{1}{a_i} \prod_{j \in inhibitors} \frac{1}{a_j^{|w_{ij}|}} \right)$$

$$\rightarrow \log(a_i) = \sum_{j \in activators} w_{ij} \log(a_j) + \sum_{j \in inhibitors} w_{ij} \log(a_j)$$

$$\rightarrow x_i = \sum_{j \in regulators} w_{ij} x_j \,, \qquad \textbf{(S16)}$$

where a represents the activity of node, and x_i is $\log(a_i)$. The signal flow in PSFC is calculated according to the topological order of the nodes in the signaling network. In other words, the computation of a_i depends on the upstream regulators, a_j, that are computed earlier. The following is matrix notation of equation (**S16**).

$$x(t+1) = Wx(t) \,, \qquad \textbf{(S17)}$$

where t is the order defined by the topological sort. Note that t of $x(t)$ represents simulation time in SP algorithm, and PSF has various options for loop handling [4]. Therefore, the calculation results of equation (**S17**) in PSFC are different from those of SP.

S1.4. Gaussian Smoothing (GS)

Gaussian smoothing (GS) is a family of algorithms [6] such as ‘label propagation’ [7,8] and ‘signed network propagation’ [9]. One of the major differences between SP and these algorithms is the consideration of direction and sign in network topology. Protein-protein interaction (PPI) networks, which are non-directional (i.e., symmetric), unsigned networks, were used for predicting gene functions [8] or the effects of mutations [10,11]. Gene correlation graphs that are non-directional, signed networks were constructed for finding differential gene expressions or DNA copy number variations [9]. However, SP deals with signaling networks, which are directed, signed networks.

GS algorithms ‘smoothe’ a network by minimizing or maximizing difference between the states of the connected nodes in the network. Interestingly, the mathematical form of GS is almost the same as the exact solution form of SP if a given network is symmetric (i.e., adjacency matrix, $A^T=A$) [7,9].

$$\Omega(x)=\beta\sum_{(i,j)\in links}\left|W_{ij}\right|\left(x_i-\mathrm{sgn}(W_{ij})x_j\right)^2+\gamma\sum_{i\in nodes}\left(x_i-b_i\right)^2 \quad \textbf{(S18)}$$

$$\rightarrow\ \arg\min_x \Omega(x)=\beta x^T W x+\gamma(x-b)^T(x-b)$$

$$\rightarrow\ \frac{\partial\Omega(x)}{\partial x}=\beta(W+W^T)x+2\gamma(x-b)=0 \quad \textbf{(S19)}$$

$$\rightarrow\ x^*=\left(I-\alpha W\right)^{-1}\cdot b\,,$$

where $\alpha=-\beta/\gamma$, and W, x, b, are the same as those explained in the main manuscript. However, the solutions are different between SP and GS for asymmetric networks, because the transpose of weight matrix in equation (**S19**) is not equal to itself (i.e., $W^T\neq W$). Thus, for asymmetric networks, the solution of equation (**S18**) becomes the following form.

$$x^*=\left(I-\frac{\alpha}{2}(W+W^T)\right)^{-1}\cdot b\,.$$

Example 2. Consider two-node negative feedback loops, where the sources of negative links are different.

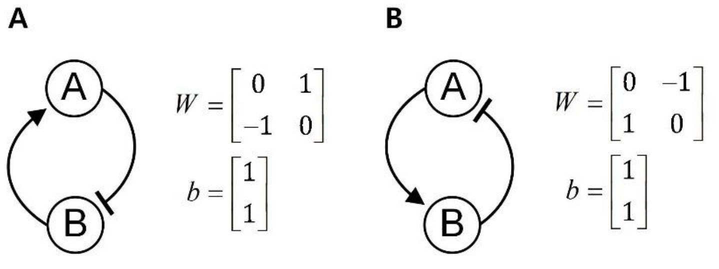

$$\left|W_{AB}\right|\left(A+B\right)^2+\left|W_{BA}\right|\left(B-A\right)^2 \qquad \left|W_{AB}\right|\left(A-B\right)^2+\left|W_{BA}\right|\left(B+A\right)^2$$

$$\rightarrow\left|W_{AB}\right|\left(A+B\right)^2+\left|W_{BA}\right|\left(A-B\right)^2 \qquad \rightarrow\left|W_{BA}\right|\left(A+B\right)^2+\left|W_{AB}\right|\left(A-B\right)^2$$

The computation results of the two-node negative feedback loops (α=-β/γ=0.5)			
Gaussian smoothing		Signal propagation	
Negative feedback loop (A)	Negative feedback loop (B)	Negative feedback loop (A)	Negative feedback loop (B)
$x^* = \begin{bmatrix} x_A \\ x_B \end{bmatrix} = \begin{bmatrix} 1 \\ 1 \end{bmatrix}$	$x^* = \begin{bmatrix} x_A \\ x_B \end{bmatrix} = \begin{bmatrix} 1 \\ 1 \end{bmatrix}$	$x^* = \begin{bmatrix} x_A \\ x_B \end{bmatrix} = \begin{bmatrix} 0.2 \\ 0.6 \end{bmatrix}$	$x^* = \begin{bmatrix} x_A \\ x_B \end{bmatrix} = \begin{bmatrix} 0.6 \\ 0.2 \end{bmatrix}$

GS cannot distinguish the directions of the links due to the quadratic term to measure the difference between the state variables in equation (**S18**), resulting in the same solutions if W_{AB} and W_{BA} are assigned the same value. However, SP has different solutions, distinguishing the directions: the activity of the target node of the negative link is smaller than that of the source node in SP.

Example 3. Consider three-node positive cascades.

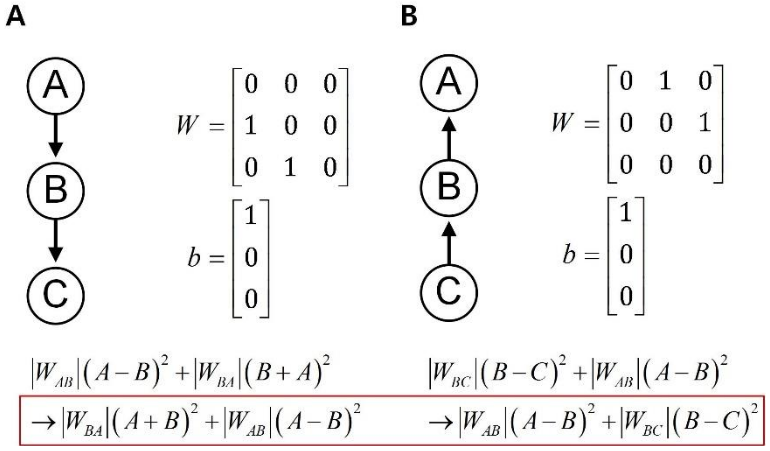

The computation results of the two-node negative feedback loops (α=-β/γ=0.5)			
Gaussian smoothing		Signal propagation	
Positive cascade (A)	Positive cascade (B)	Positive cascade (A)	Positive cascade (B)
$x^* = \begin{bmatrix} x_A \\ x_B \\ x_C \end{bmatrix} = \begin{bmatrix} 1.5 \\ 1.0 \\ 0.5 \end{bmatrix}$	$x^* = \begin{bmatrix} x_A \\ x_B \\ x_C \end{bmatrix} = \begin{bmatrix} 1.5 \\ 1.0 \\ 0.5 \end{bmatrix}$	$x^* = \begin{bmatrix} x_A \\ x_B \\ x_C \end{bmatrix} = \begin{bmatrix} 0.5 \\ 0.25 \\ 0.125 \end{bmatrix}$	$x^* = \begin{bmatrix} x_A \\ x_B \\ x_C \end{bmatrix} = \begin{bmatrix} 0.5 \\ 0.0 \\ 0.0 \end{bmatrix}$

GS ignores the directions for these different positive cascades, as in the example of the negative feedback loop, whereas the results of both cases are different in SP. Note that nodes A and B have non-zero values for the positive cascade (B) in GS despite there is no path from node A to node C. This aspect of GS might not be adequate for directed networks such as biological signaling networks.

II. Supplementary Figures

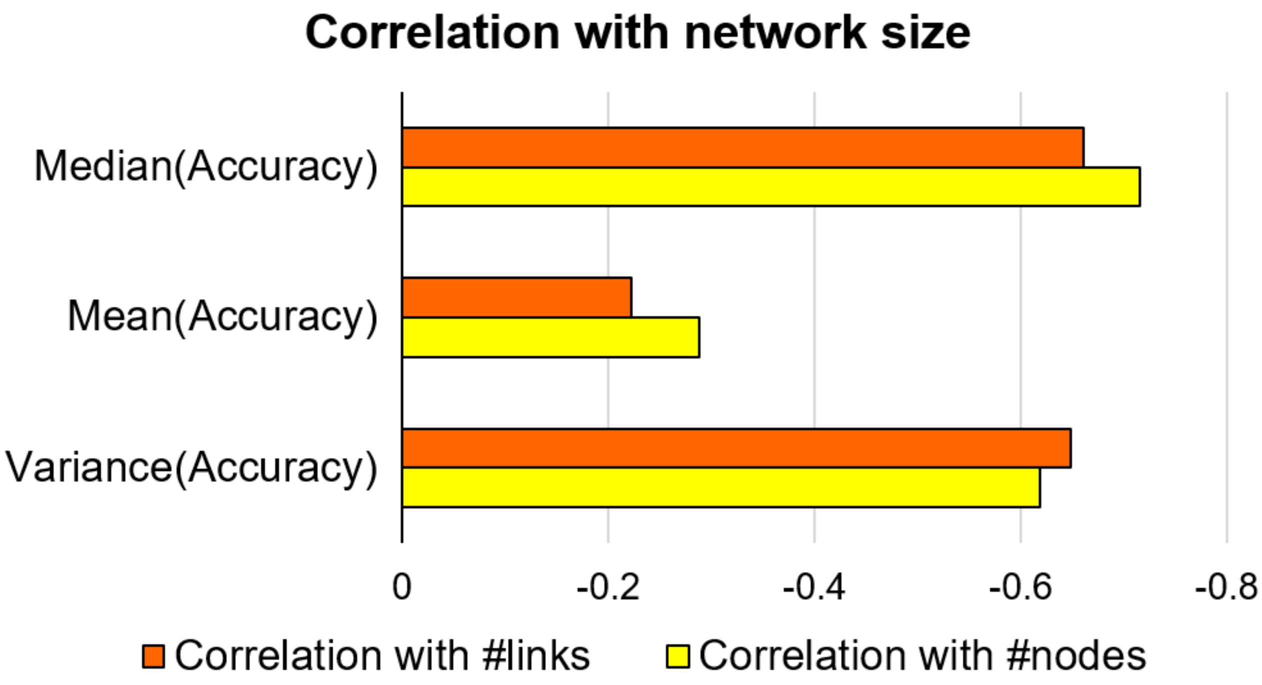

Supplementary Figure S1. Correlation between accuracy and network size. Negative correlation between the statistics of the accuracy (median, mean, or variance) and network size (number of links or number of nodes) for the six datasets. Pearson correlation coefficient was calculated for each pair.

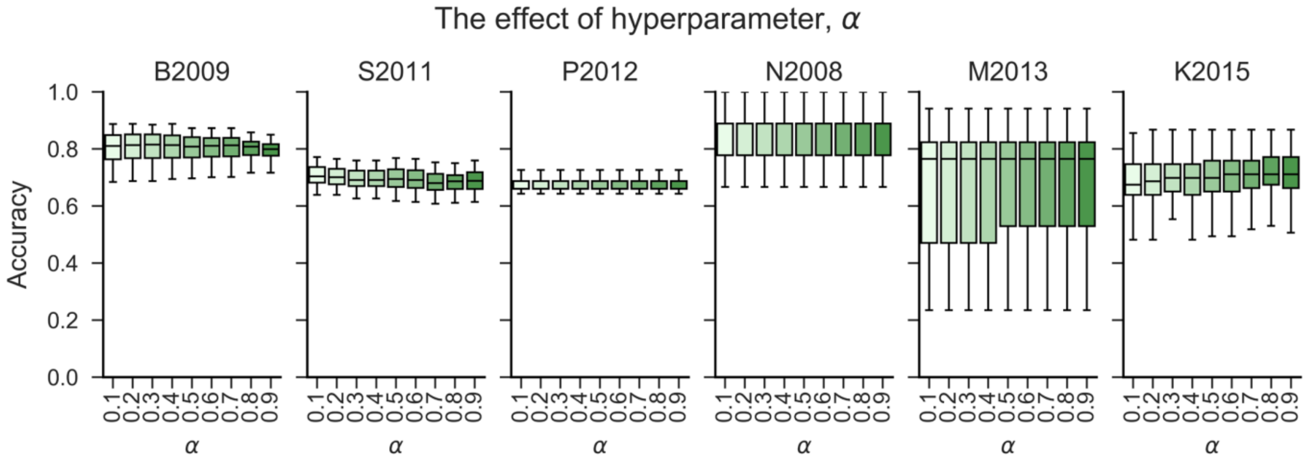

Supplementary Figure S2. The effect of hyperparameter, α. The predictive power of SP algorithm was not substantially affected by the hyperparameter, *α*.

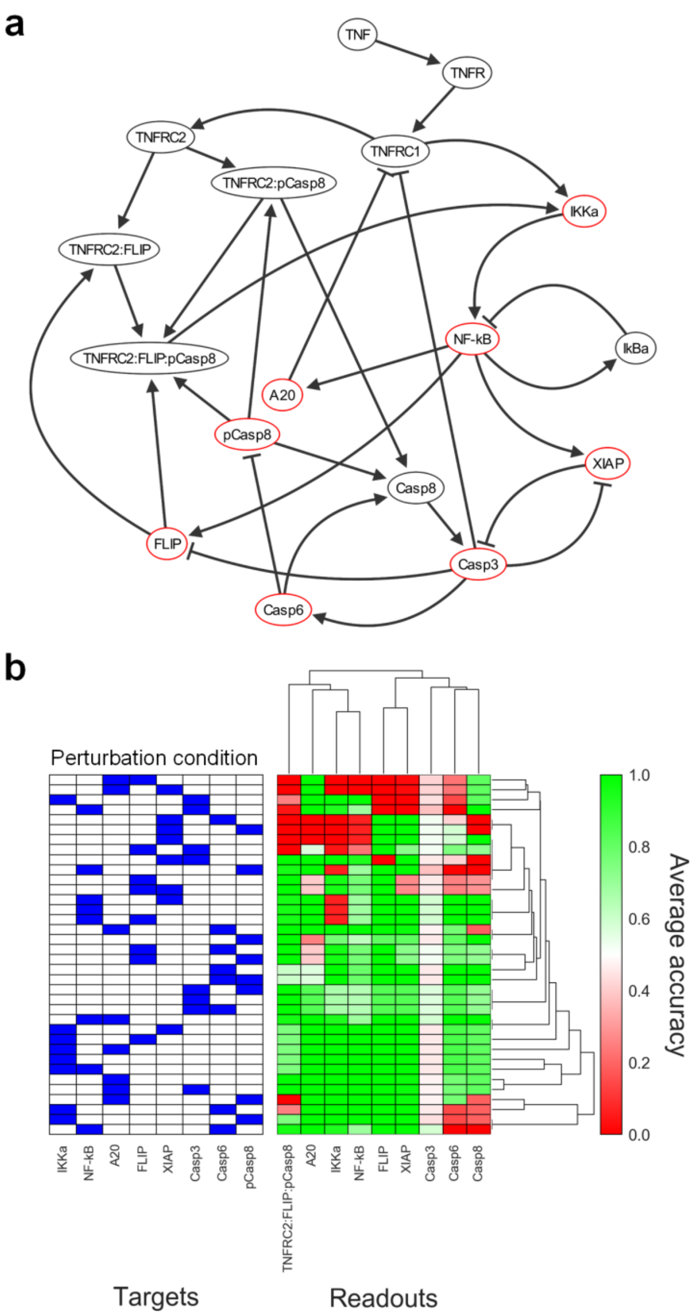

Supplementary Figure S3. Network structure and hierarchical clustering result of S2011.

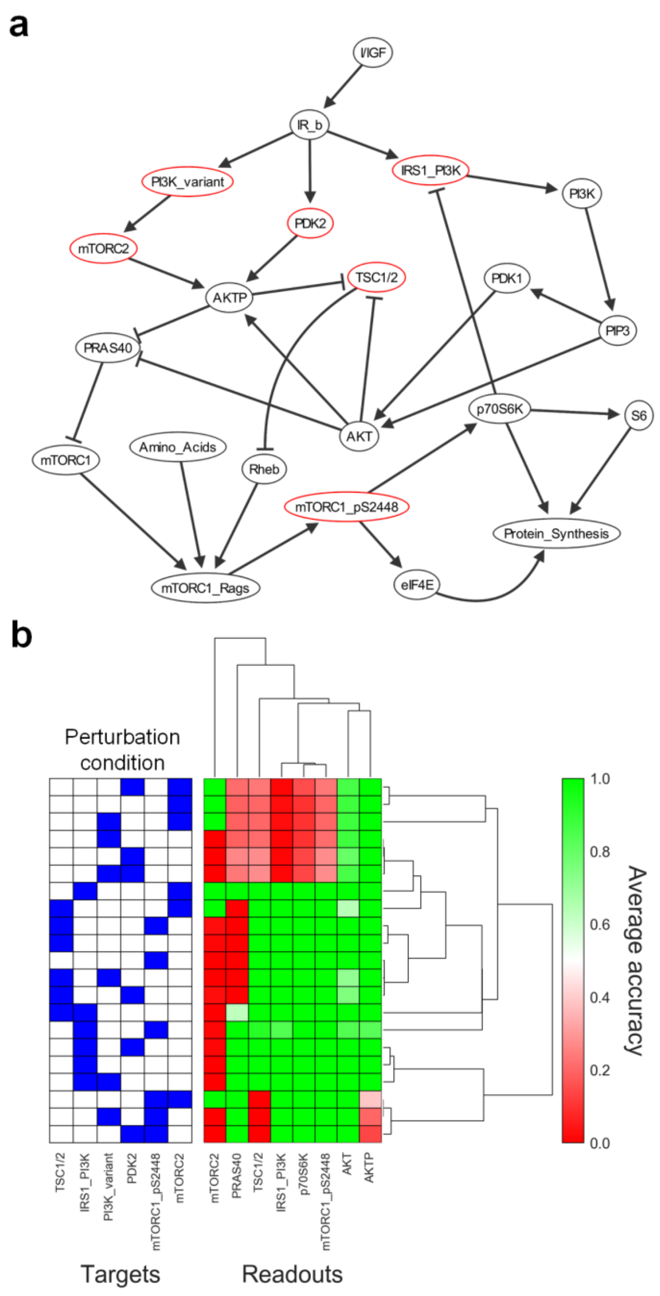

Supplementary Figure S4. Network structure and hierarchical clustering result of P2012.

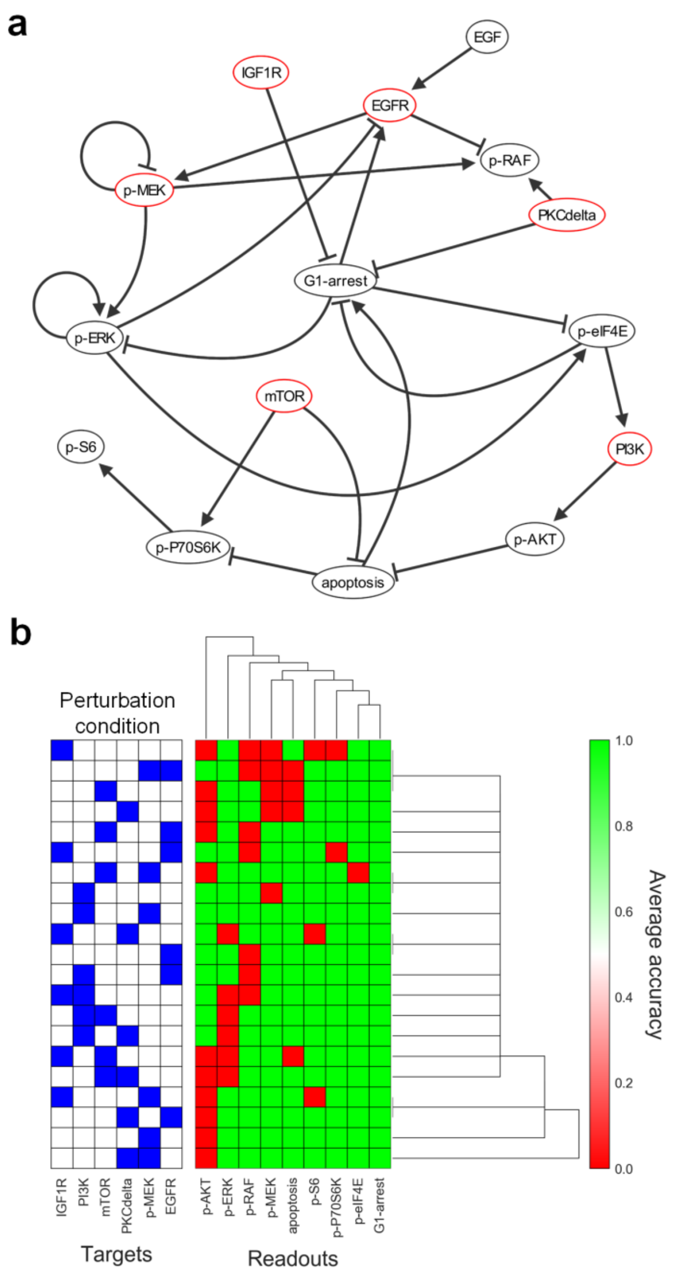

Supplementary Figure S5. Network structure and hierarchical clustering result of N2008.

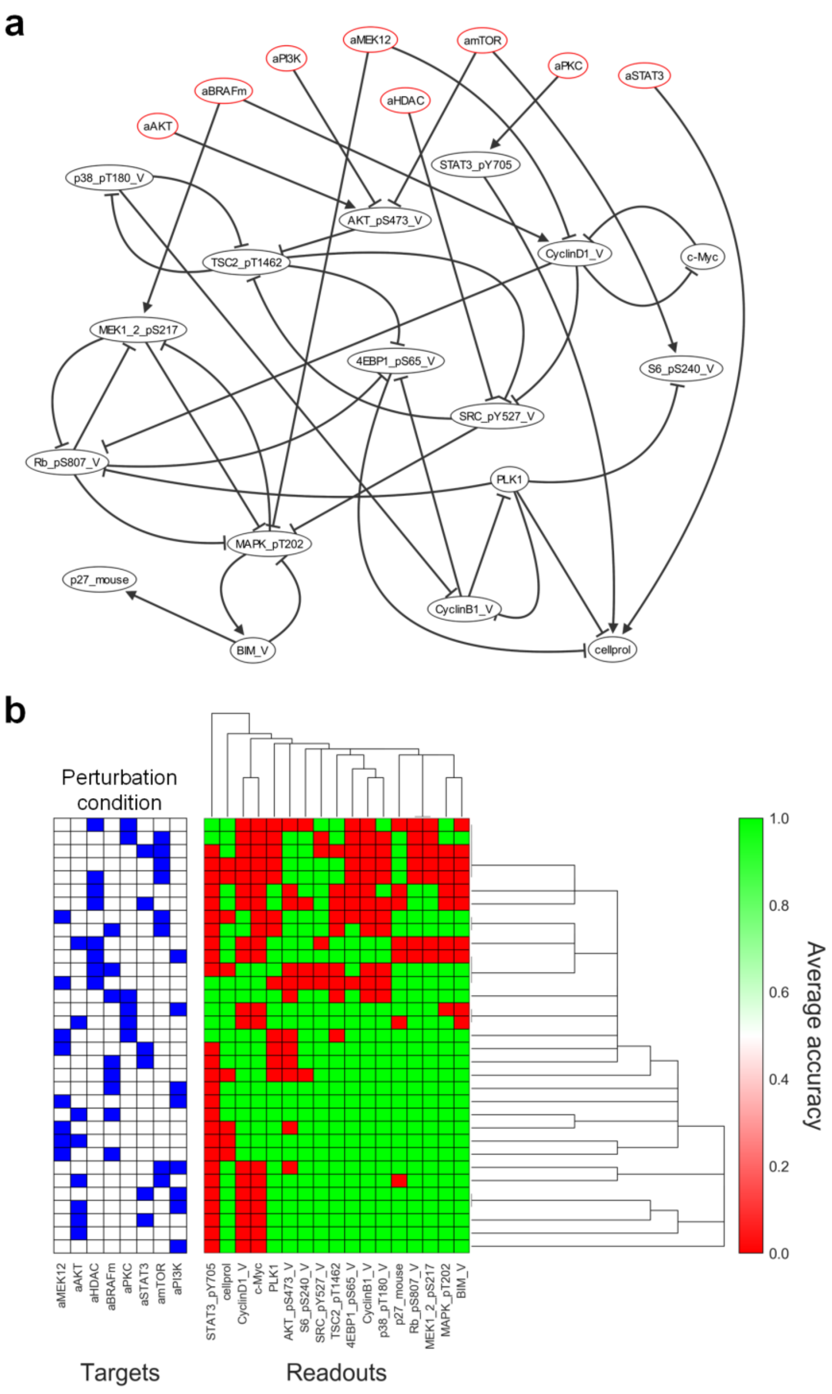

Supplementary Figure S6. Network structure and hierarchical clustering result of M2013.

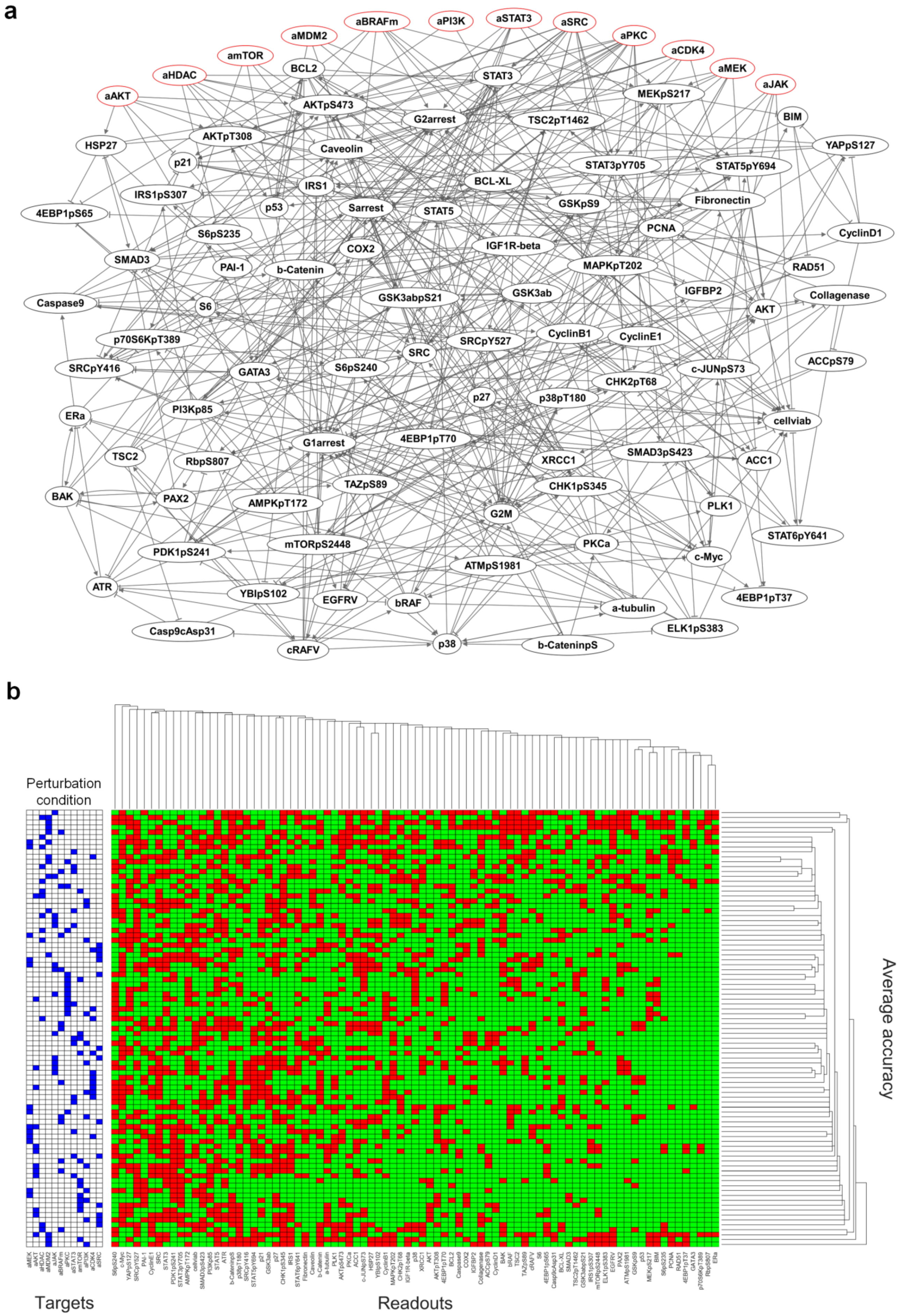

Supplementary Figure S7. Network structure and hierarchical clustering result of K2015.

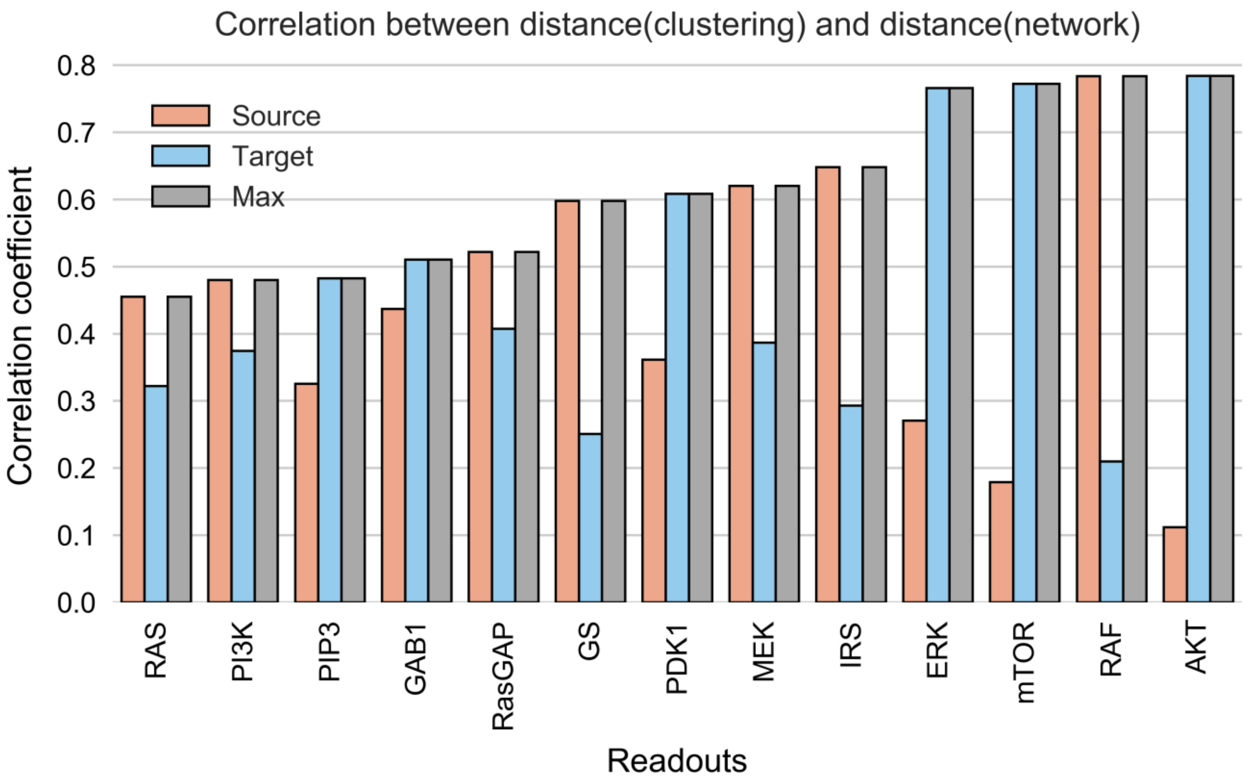

Supplementary Figure S8. Correlation between the distances in network structure and the distances in dendrogram of hierarchical clustering for B2009. Source: shortest path distances between the node and the other nodes in the network when the node is source; Target: shortest path distances between the node and the other nodes in the network when the node is target; Max: the maximum between correlation coefficients of the source and target cases.

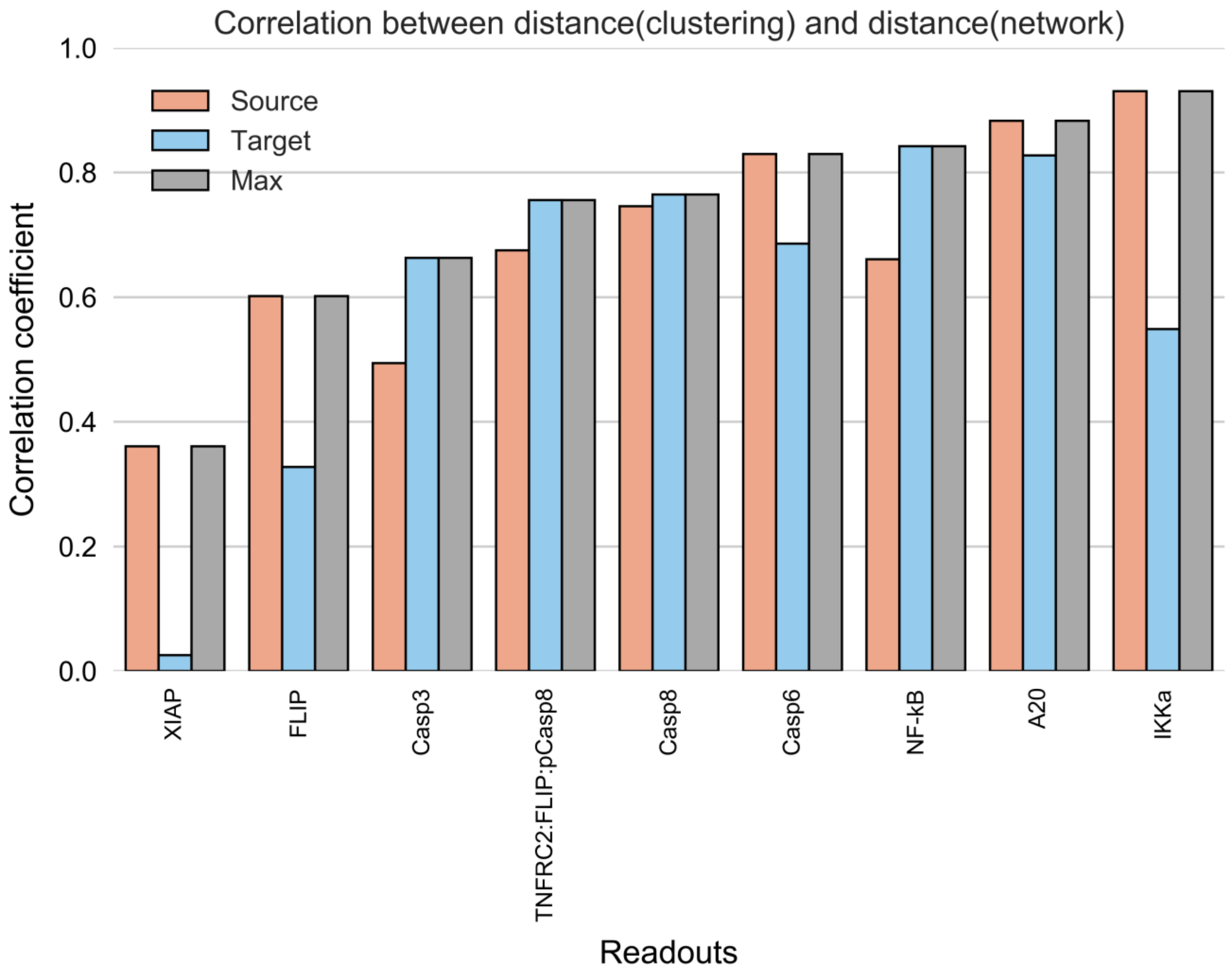

Supplementary Figure S9. Correlation between the distances in network structure and the distances in dendrogram of hierarchical clustering for S2011. Source, Target, and Max: same as Supplementary Fig. S8.

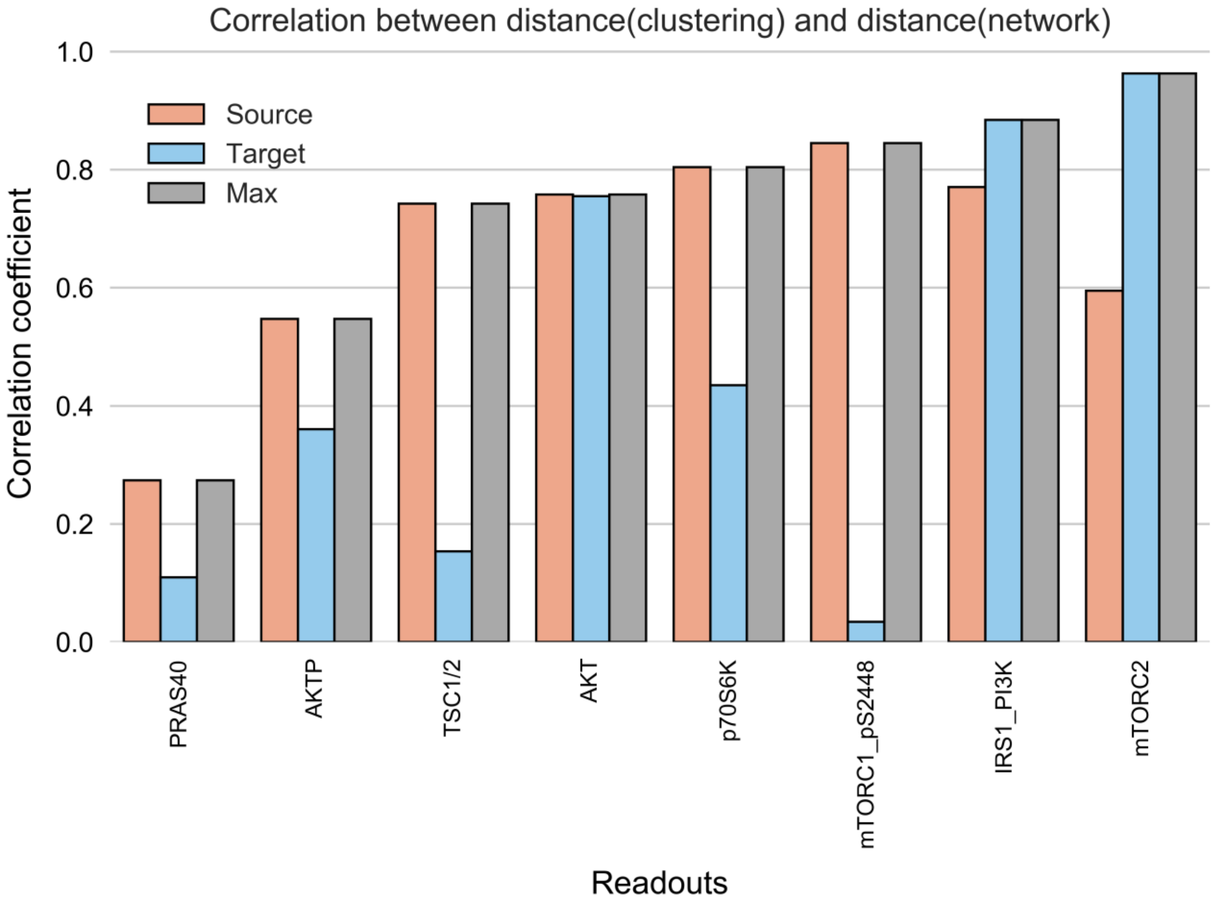

Supplementary Figure S10. Correlation between the distances in network structure and the distances in dendrogram of hierarchical clustering for P2012. Source, Target, and Max: same as Supplementary Fig. S8.

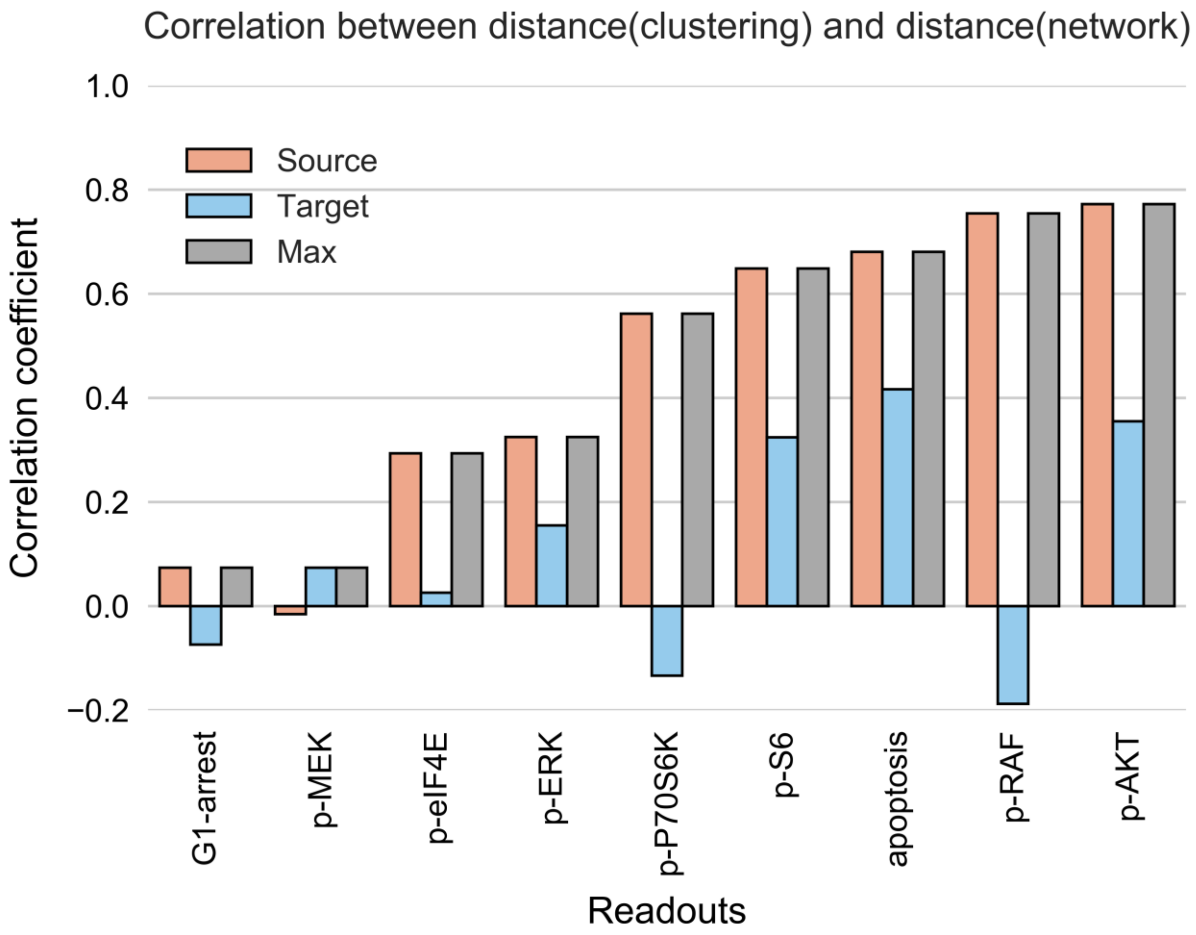

Supplementary Figure S11. Correlation between the distances in network structure and the distances in dendrogram of hierarchical clustering for N2008. Source, Target, and Max: same as Supplementary Fig. S8.

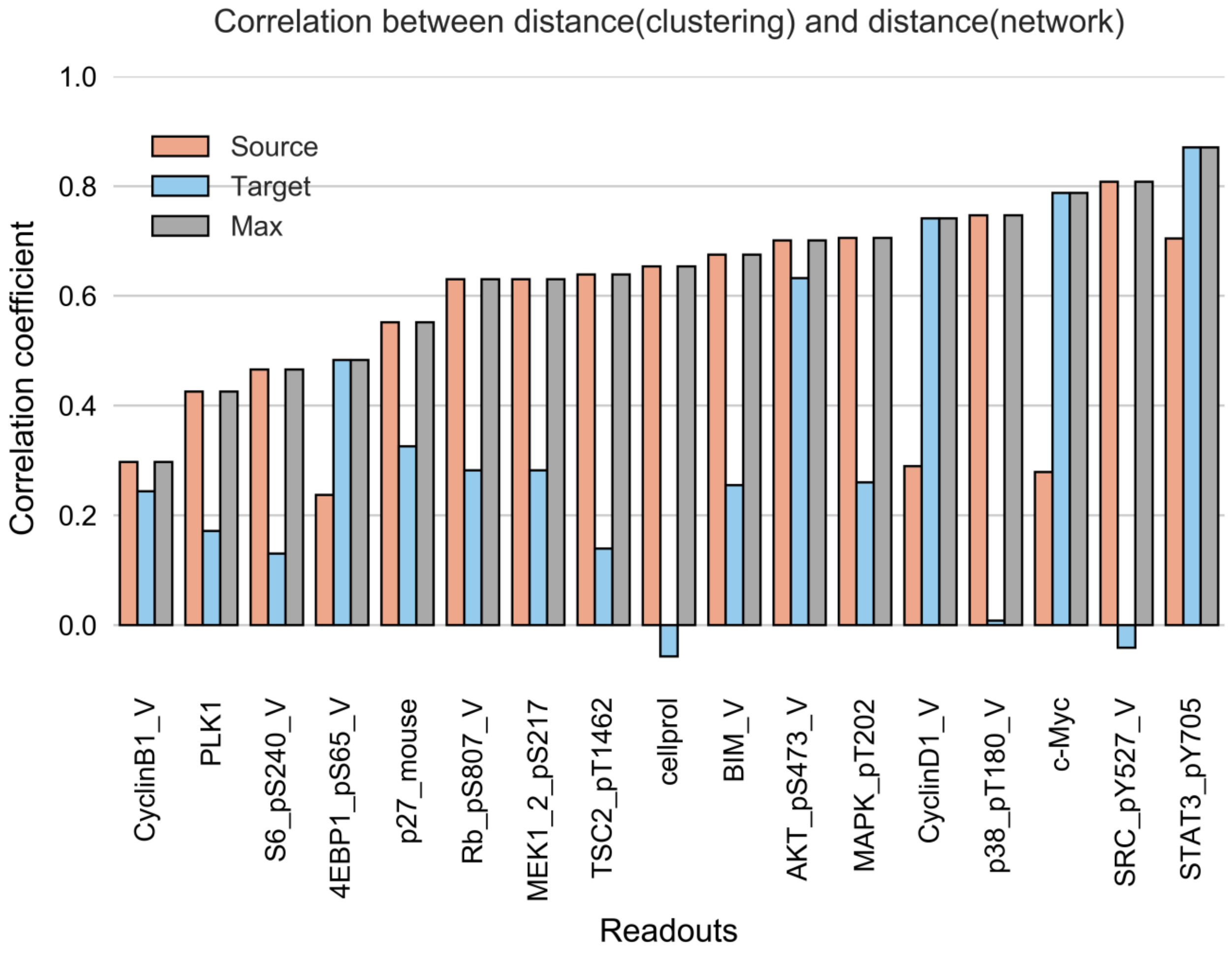

Supplementary Figure S12. Correlation between the distances in network structure and the distances in dendrogram of hierarchical clustering for M2013. Source, Target, and Max: same as Supplementary Fig. S8.

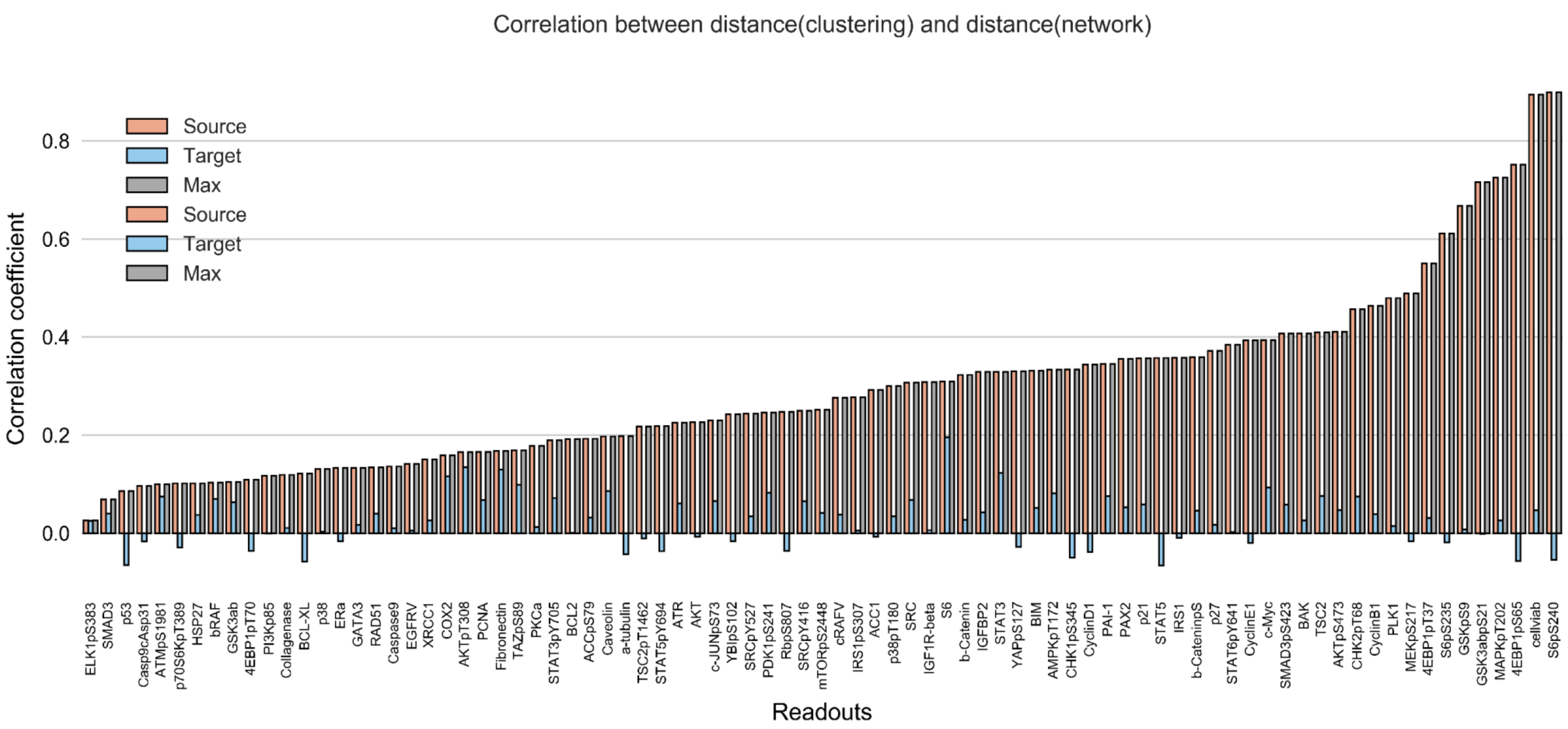

Supplementary Figure S13. Correlation between the distances in network structure and the distances in dendrogram of hierarchical clustering for K2015. Source, Target, and Max: same as Supplementary Fig. S8.

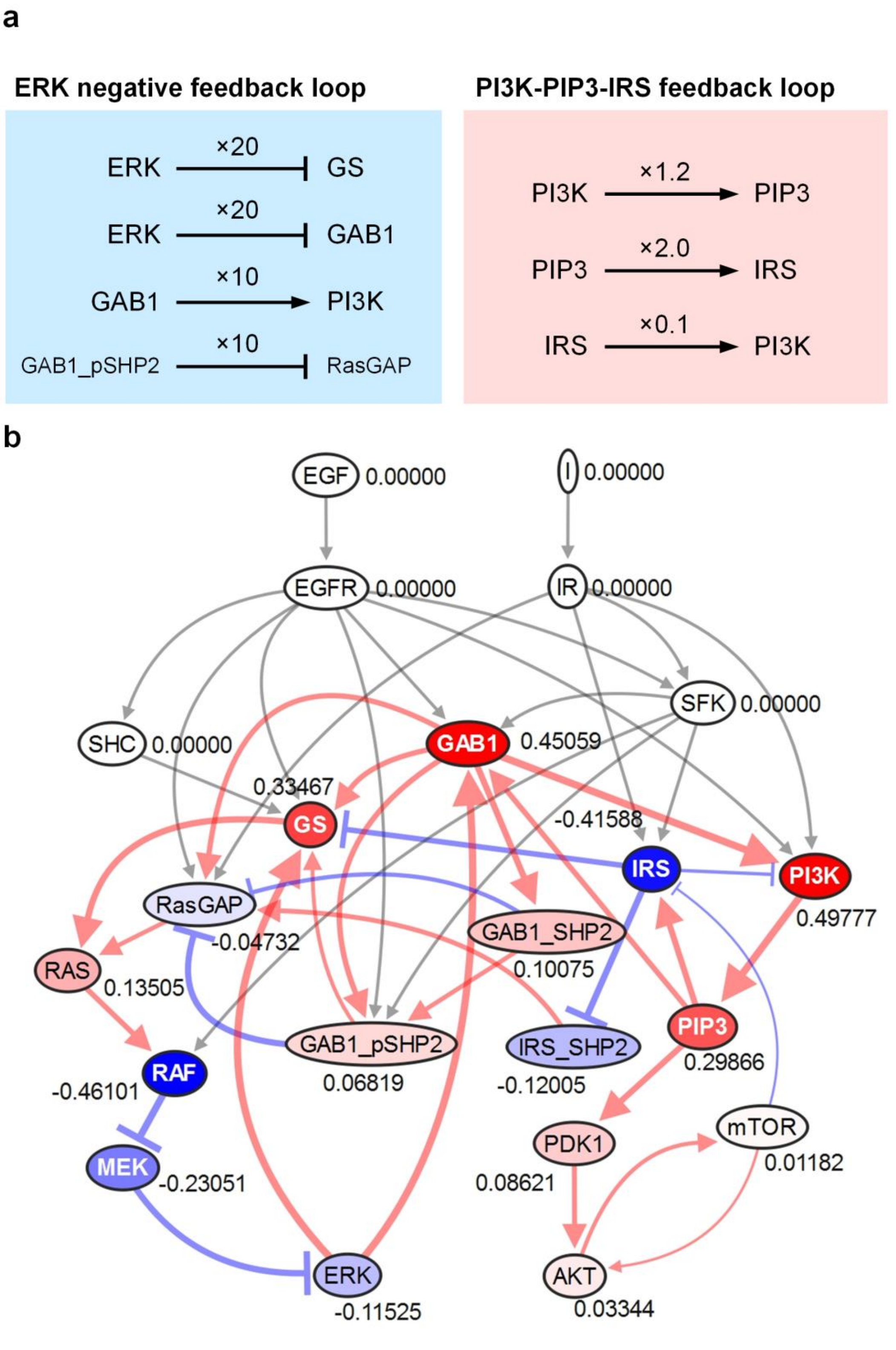

Supplementary Figure S14. Adjustment of link weights and the result of signal flow estimation. (a) Two more link weights, GAB1_pSHP2 to RasGAP and IRS to PI3K, were adjusted in addition to those of Figure 7f to achieve the agreement with the original ODE model for a certain experimental condition. **(b)** The result of signal flow estimation under the perturbation of IRS and RAF explicitly shows a subset of signal flows are amplified by the adjusted weights. All the DAC of readouts are in agreement with those of ODE (see Supplementary Fig. S15).

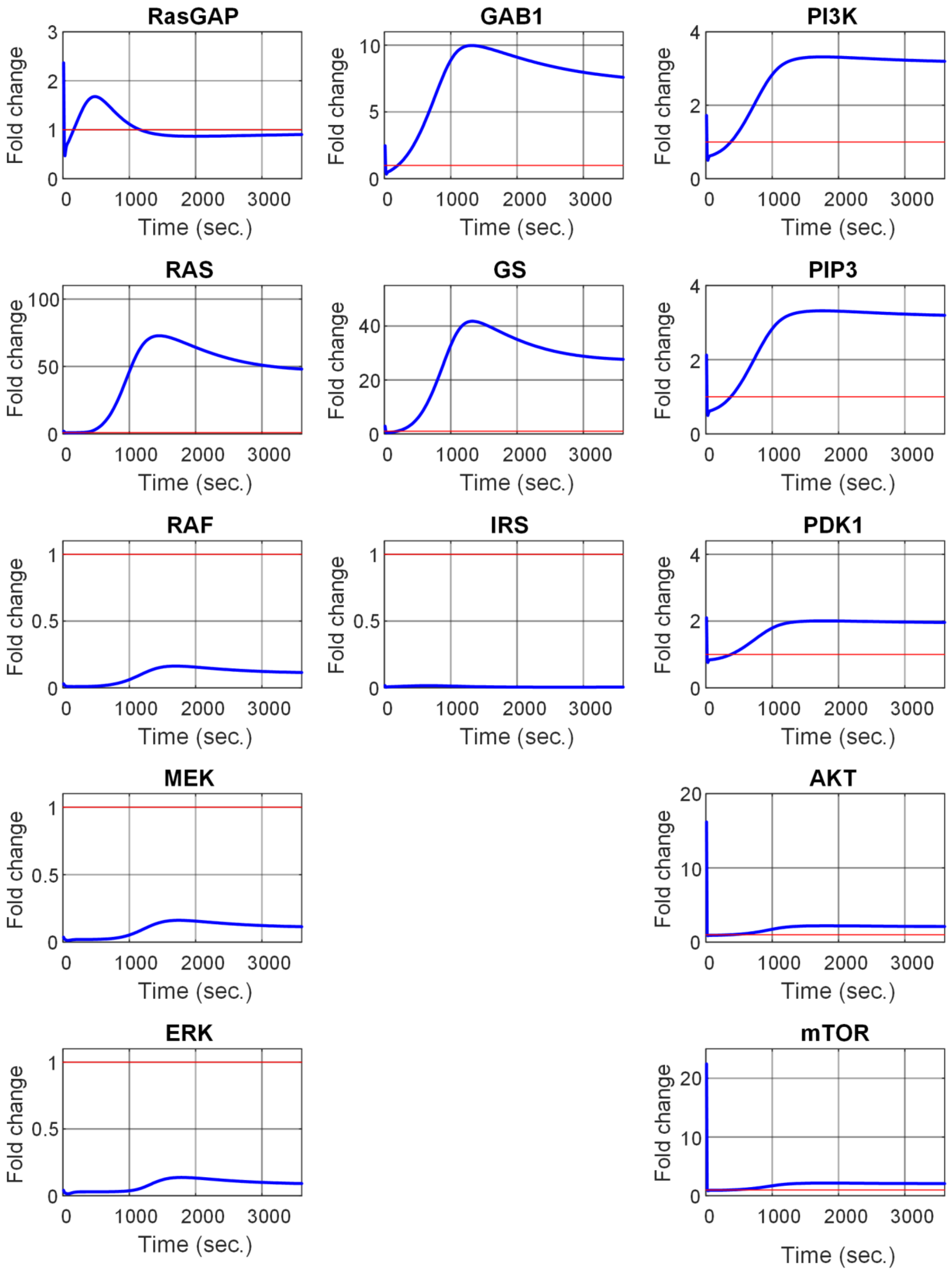

Supplementary Figure S15. The time profiles of the original ODE model under the perturbation of IRS and RAF. The original ODE model of B2009 was numerically solved under the perturbation of IRS and RAF (input stimulation: EGF=1nM, insulin=100nM; simulation time: 3600 seconds). The perturbation of IRS and RAF was implemented by decreasing the total concentration of both IRS and RAF by 99%.

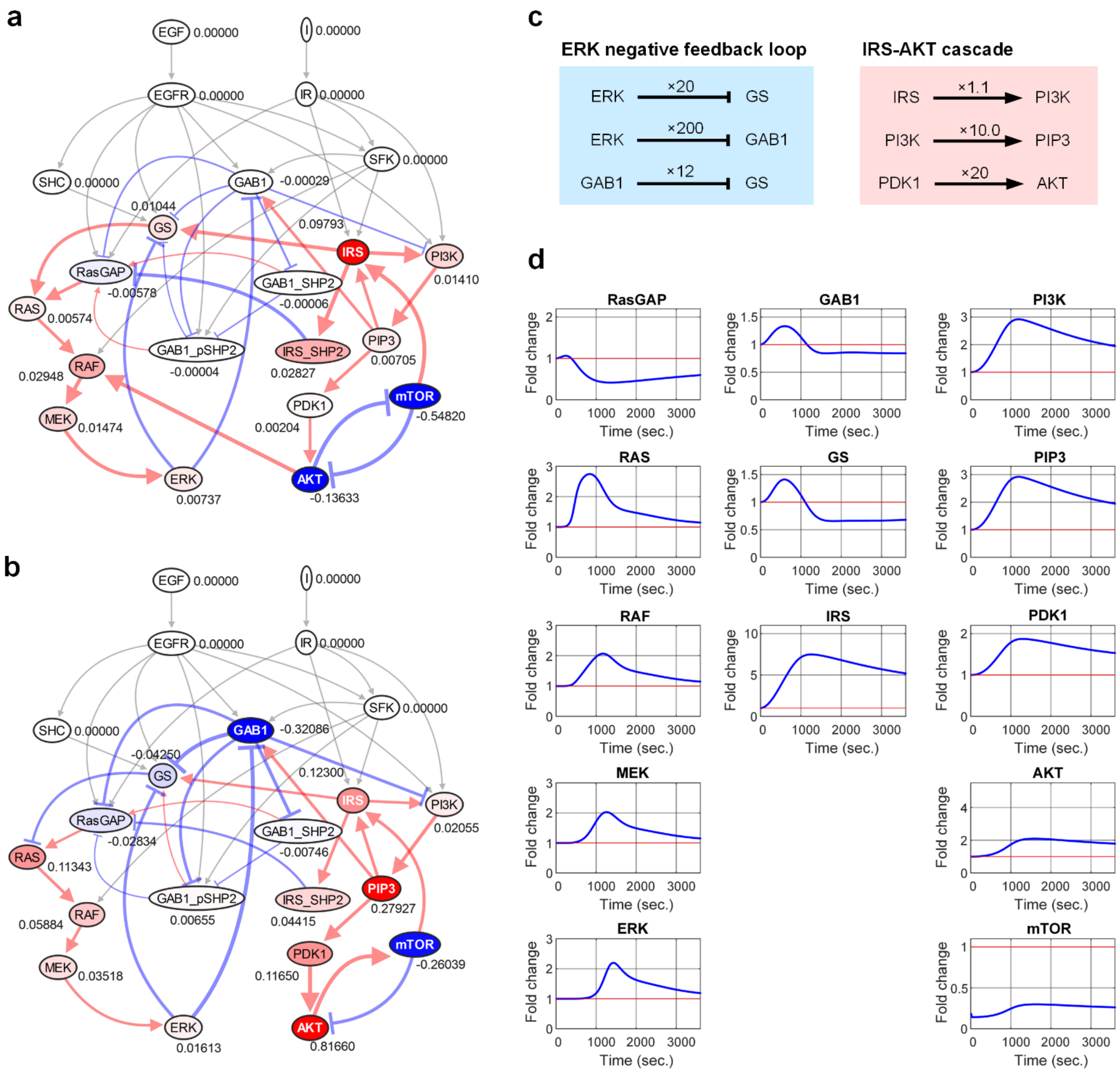

Supplementary Figure S16. An example of prediction result improved by adjusting link weights. (a) The original result of the signal flow estimation under the perturbation of mTOR in the signaling network of B2009, where the prediction result of AKT is incorrect. **(b)** The result improved by adjusting six link weights. **(c)** Six link weights that were adjusted to achieve complete agreement with the original ODE model. **(d)** The time profiles of the original ODE model under the perturbation of mTOR (input stimulation: EGF=1nM, insulin=100nM; simulation time: 3600 seconds). The perturbation of mTOR was implemented by decreasing the total concentration of mTOR by 90%.

III. Supplementary References

1 Feiglin, A. *et al.* Static network structure can be used to model the phenotypic effects of perturbations in regulatory networks. *Bioinformatics* **28**, 2811-2818, doi:10.1093/bioinformatics/bts517 (2012).

2 Stanley, R. P. *Algebraic combinatorics : walks, trees, tableaux, and more*. (Springer, 2013).

3 Arakelyan, A., Aslanyan, L. & Boyajyan, A. High-throughput gene expression analysis concepts and applications. *Sequence and Genome Analysis II-Bacteria, Viruses and Metabolic Pathways* (2013).

4 Nersisyan, L., Johnson, G., Riel-Mehan, M., Pico, A. & Arakelyan, A. PSFC: a Pathway Signal Flow Calculator App for Cytoscape. *F1000Res* **4**, 480, doi:10.12688/f1000research.6706.1 (2015).

5 Arakelyan, A., Nersisyan, L., Petrek, M., Loffler-Wirth, H. & Binder, H. Cartography of Pathway Signal Perturbations Identifies Distinct Molecular Pathomechanisms in Malignant and Chronic Lung Diseases. *Front Genet* **7**, 79, doi:10.3389/fgene.2016.00079 (2016).

6 Wang, P. I. & Marcotte, E. M. It's the machine that matters: Predicting gene function and phenotype from protein networks. *Journal of Proteomics* **73**, 2277-2289, doi:10.1016/j.jprot.2010.07.005 (2010).

7 Zhou, D., Bousquet, O., Lal, T. N., Weston, J. & Schölkopf, B. Learning with local and global consistency. *Advances in Neural Information Processing Systems 16* **1**, 595-602, doi:citeulike-article-id:922481 (2004).

8 Mostafavi, S., Ray, D., Warde-Farley, D., Grouios, C. & Morris, Q. GeneMANIA: a real-time multiple association network integration algorithm for predicting gene function. *Genome Biol* **9 Suppl 1**, S4, doi:10.1186/gb-2008-9-s1-s4 (2008).

9 Zhang, W., Johnson, N., Wu, B. & Kuang, R. in *Proceedings of the ACM Conference on Bioinformatics, Computational Biology and Biomedicine - BCB '12* 337-344 (ACM Press, New York, New York, USA, 2012).

10 Cho, A. *et al.* MUFFINN: cancer gene discovery via network analysis of somatic mutation data. *Genome Biol* **17**, 129, doi:10.1186/s13059-016-0989-x (2016).

11 Shin, D., Lee, J., Gong, J. R. & Cho, K. H. Percolation transition of cooperative mutational effects in colorectal tumorigenesis. *Nat Commun* **8**, 1270, doi:10.1038/s41467-017-01171-6 (2017).
